# Endothelial Nitric Oxide Synthase G894T Polymorphism Associates with Disease Severity in Puumala Hantavirus Infection

**DOI:** 10.1371/journal.pone.0142872

**Published:** 2015-11-11

**Authors:** Sirpa Koskela, Outi Laine, Satu Mäkelä, Tanja Pessi, Sari Tuomisto, Heini Huhtala, Pekka J. Karhunen, Ilkka Pörsti, Jukka Mustonen

**Affiliations:** 1 Department of Internal Medicine, Tampere University Hospital, Tampere, Finland; 2 School of Medicine, University of Tampere, Tampere, Finland; 3 Science Centre, Pirkanmaa Hospital District, Tampere, Finland; 4 School of Health Sciences, University of Tampere, Tampere, Finland; 5 Fimlab Laboratories Ltd, Tampere, Finland; Robert Bosch Hospital, GERMANY

## Abstract

**Introduction:**

Hantavirus infections are characterized by both activation and dysfunction of the endothelial cells. The underlying mechanisms of the disease pathogenesis are not fully understood. Here we tested the hypothesis whether the polymorphisms of endothelial nitric oxide synthase, eNOS G894T, and inducible nitric oxide synthase, iNOS G2087A, are associated with the severity of acute Puumala hantavirus (PUUV) infection.

**Patients and Methods:**

Hospitalized patients (n = 172) with serologically verified PUUV infection were examined. Clinical and laboratory variables reflecting disease severity were determined. The polymorphisms of eNOS G894T (Glu298Asp, rs1799983) and iNOS G2087A (Ser608Leu, rs2297518) were genotyped.

**Results:**

The rare eNOS G894T genotype was associated with the severity of acute kidney injury (AKI). The non-carriers of G-allele (TT-homozygotes) had higher maximum level of serum creatinine than the carriers of G-allele (GT-heterozygotes and GG-homozygotes; median 326, range 102–1041 vs. median 175, range 51–1499 μmol/l; p = 0.018, respectively). The length of hospital stay was longer in the non-carriers of G-allele than in G-allele carriers (median 8, range 3–14 vs. median 6, range 2–15 days; p = 0.032). The rare A-allele carriers (*i*.*e*. AA-homozygotes and GA-heterozygotes) of iNOS G2087A had lower minimum systolic and diastolic blood pressure than the non-carriers of A-allele (median 110, range 74–170 vs.116, range 86–162 mmHg, p = 0.019, and median 68, range 40–90 vs. 72, range 48–100 mmHg; p = 0.003, respectively).

**Conclusions:**

Patients with the TT-homozygous genotype of eNOS G894T had more severe PUUV-induced AKI than the other genotypes. The eNOS G894T polymorphism may play role in the endothelial dysfunction observed during acute PUUV infection.

## Introduction

Hantaviruses cause two clinical syndromes in humans, the haemorrhagic fever with renal syndrome (HFRS) in Europe and Asia, and hantavirus cardiopulmonary syndrome (HCPS) in the Americas. Puumala hantavirus (PUUV) is the most common hantavirus causing HFRS in Europe [1]. The main characteristics of PUUV-HFRS are increased capillary leakage, thrombocytopenia and acute kidney injury (AKI). Although PUUV-HFRS has a low rate of case fatality (up to 0.4%), significant acute-phase complications as well as long-term hormonal, renal and cardiovascular consequences can occur [1, 2].

The main pathophysiological mechanisms of hantavirus infection include activation of cytokines [3, 4] and cytotoxic CD8+ T-lymphocytes [5], vascular endothelial growth factors [6, 7], and the complement system [8]. Recently discovered biomarkers that reflect PUUV-HFRS disease severity include pentraxin-3, indoleamine 2,3-dioxygenase, plasma cell-free DNA, soluble urokinase-type plasminogen activator and GATA-3 [9]. Host genetic factors also influence the outcome of acute PUUV infection. In the Finnish population, individuals with Human Leukocyte Antigen (HLA) alleles B8, C4A*Q0 and DRB1*0301 are more prone to have a severe form of PUUV infection [10, 11]. Also polymorphisms of the cytokines tumor necrosis factor alpha (TNF-α), interleukin-1 (IL-1) and IL-1 receptor antagonist impact on the clinical severity of PUUV-HFRS [10, 12]. Likewise, genetic polymorphisms of plasminogen activator inhibitor, the main physiological regulator of fibrinolysis, and platelet glycoprotein 1a, associate with severe PUUV infection [13].

Increased nitric oxide (NO) levels induced by elevated TNF-α concentrations have been suggested to participate in the pathogenesis of hantaviral infections [14, 15, 16]. Elevated concentrations of NO correlate with increased serum creatinine value and hypotension, and inversely correlate with platelet count in patients with acute PUUV infection [15]. According to a Swedish study, NO has antiviral effects on hantaviruses by inhibiting viral replication at the early phase of infection [17]. Endothelial nitric oxide synthase (eNOS), and inducible nitric oxide synthase (iNOS) that can be induced in a variety of cell types, are the key enzymes catalysing NO synthesis [18].

A widely explored polymorphism of the eNOS gene, the G894T (rs1799983) polymorphism encoded by the NOS3 gene in chromosome 7, has been linked to increased risk of coronary artery disease (CAD), myocardial infarction, coronary spasms, hypertension, and ischemic stroke [19–24]. Recent data suggest that the T-allele of G894T polymorphism is also associated with increased susceptibility to and risk of end-stage renal disease (ESRD) [25], and also with earlier onset age of ESRD in males with autosomal dominant polycystic disease [26]. The iNOS, encoded by the NOS2 gene in chromosome 17, is expressed in macrophages, neutrophils and hepatocytes as a host immune response to cytokines. The G2087A (rs2927518) polymorphism of iNOS has been implicated in a variety of diseases, including inflammatory bowel disease, gastric cancer, migraine with aura, septic shock, and non-Hodgkin lymphoma [27–30]. So far there is no evidence whether the NOS polymorphisms influence the clinical course of hantaviral infections.

We aimed to study the influence of the above polymorphisms that have the potential to affect endothelial and vascular function, eNOS G894T (Glu298Asp, rs1799983) and iNOS G2087A (Ser608Leu, rs2297518), on disease severity in patients with acute PUUV infection. We sought to determine whether the genetic variations within the genes of NOS3 and NOS2 could contribute to individual differences in the outcome of acute PUUV infection.

## Material and Methods

### Ethics statement

The study was carried out at Tampere University Hospital and University of Tampere, School of Medicine. All patients were recruited and enrolled after providing a written informed consent. In addition, informed verbal consent was obtained from the guardians of the minors. Blood samples of the minors were collected before the current Medical Reseach Act was valid in Finland. The Ethics Committee of Tampere University Hospital approved the study protocol and consent procedure according to the ethical principles at the time of the study. The study was conducted according to the principles expressed in the Declaration of Helsinki.

### Patients

The study cohort consisted of 172 prospectively collected, consecutive hospitalized patients with serologically confirmed acute PUUV infection. The collection of clinical data including routine laboratory measurements has been described in detail elsewhere [13].

All patients came from the Pirkanmaa area and were hospitalized in Tampere University Hospital, Finland for the median time of six days (range 2–15) during the period from September 1997 to February 2009. The median age of the patients was 40 years (ranging from 15 to 74 years), and 119 were males (69%). The concomitant diseases of the study group were arterial hypertension (n = 12), dyslipidemia (n = 7), CAD (n = 5), bronchial asthma (n = 6), atrial fibrillation (n = 3) and rheumatoid arthritis (n = 3). There were also patients with celiac disease, inflammatory bowel disease, valvular heart disease or neurological disease (n = 2 for each).

### Definition of AKI

AKI was defined according to Kidney Disease: Improving Global Outcomes (KDIGO) criteria as an increase in plasma creatinine ≥1.5 times baseline, which was presumed to have occurred within the prior seven days [31]. The upper limits of the reference values for plasma creatinine (women 90 μmol/l and men 100 μmol/l) were taken as baseline levels. Thus, plasma creatinine ≥135 μmol/l in women, and ≥150 μmol/l in men was defined as AKI.

### Genotyping

DNA was extracted from whole blood using a commercially available kit (QIAGEN Inc., Hilden, Germany). The gene polymorphisms of eNOS G894T (Glu298Asp, rs1799983) and iNOS G2087A (Ser608Leu, rs2297518) were genotyped with TaqMan® SNP Genotyping Assay (Life Technologies Ltd, Carlsbad, CA, USA) under standard conditions using the ABI Prism 7900HT Sequence Detection System (Taqman, Applied Biosystems, Foster City, CA, USA). Reaction volume used was 5 μl and it was prepared with TaqMan Genotyping MasterMix (Life Technologies Ltd, Carlsbad, CA, USA). Amplification data were analyzed with SDS 2.2 software (Taqman, Applied Biosystems, Foster City, CA, USA). The distributions of all SNPs did not deviate from the Hardy–Weinberg equation. The genotyping was successful in 167 of 172 patients (97%) for eNOS and in 166 patients (96.5%) for iNOS.

### Statistical analysis

The highest and lowest values of continuous variables measured during acute PUUV infection were designated as maximum or minimum values. In order to describe the data, medians and ranges were given for skewed continuous variables and numbers for categorical variables. The allele frequencies were calculated, and the patients were grouped into carriers (including both homozygotes and heterozygotes) and non-carriers of specific alleles. Differences in the clinical severity of PUUV infection between groups were tested using Mann-Whitney U-test or Kruskal-Wallis test for numerical data and χ^2^-test or Fisher`s exact test for categorical data, as appropriate. All p-values were two-tailed, and the statistical significance was considered at 0.05. Statistical analyses were performed using IBM SPSS software version 21.

## Results

### Clinical and laboratory findings

The clinical and laboratory findings of the 172 patients with acute PUUV infection are shown in Table 1. All patients suffered from serologically verified [32] and clinically typical PUUV infection, and they were examined and hospitalized during the acute phase of illness. Three patients (2%) were in clinical shock on admission to the hospital. AKI was found in 96 (56%) patients. Seven patients (4%) needed hemodialysis treatment during hospitalization. All patients recovered.

**Table 1:** The clinical and laboratory findings in 172 patients with acute Puumala hantavirus infection.

Clinical or laboratory variable	Median	Range
Days from the onset of fever*	4	1–14
Length of hospital stay (days)	6	2–15
Systolic blood pressure, minimum (mmHg)	113	74–170
Diastolic blood pressure, minimum (mmHg)	70	40–100
Change in weight (kg)**	2.1	0–12.0
Hematocrit, maximum	0.44	0.33–0.60
Hematocrit, minimum	0.36	0.25–0.46
Platelet count, minimum (x 10^9^/l)	62	3–238
Leukocyte count, maximum (x 10^9^/l)	10	3.9–31.2
CRP, maximum (mg/ml)	75	11–269
Creatinine, maximum (μmol/l)	185	51–1499
Interleukin-6, maximum (pg/ml)	14.5	1.3–107

Abbreviation: CRP, C-reactive protein.

Normal values: CRP < 10 mg/ml, creatinine ≤ 100 μmol/l for males and ≤ 90 μmol/l for females, platelet count 150–360 x 10^9^/l, leukocyte count 3.4–8.2 x 10^9^/l, hematocrit 0.35–0.50 for males and 0.35–0.46 for females.

*Equals to the onset of illness before the first blood test was taken.

**Change in weight during hospital stay reflects the fluid accumulation in the body during the oliguric phase.

#### Association of eNOS G894T (Glu298Asp, rs1799983) polymorphism with clinical and laboratory findings

**Fig 1:**
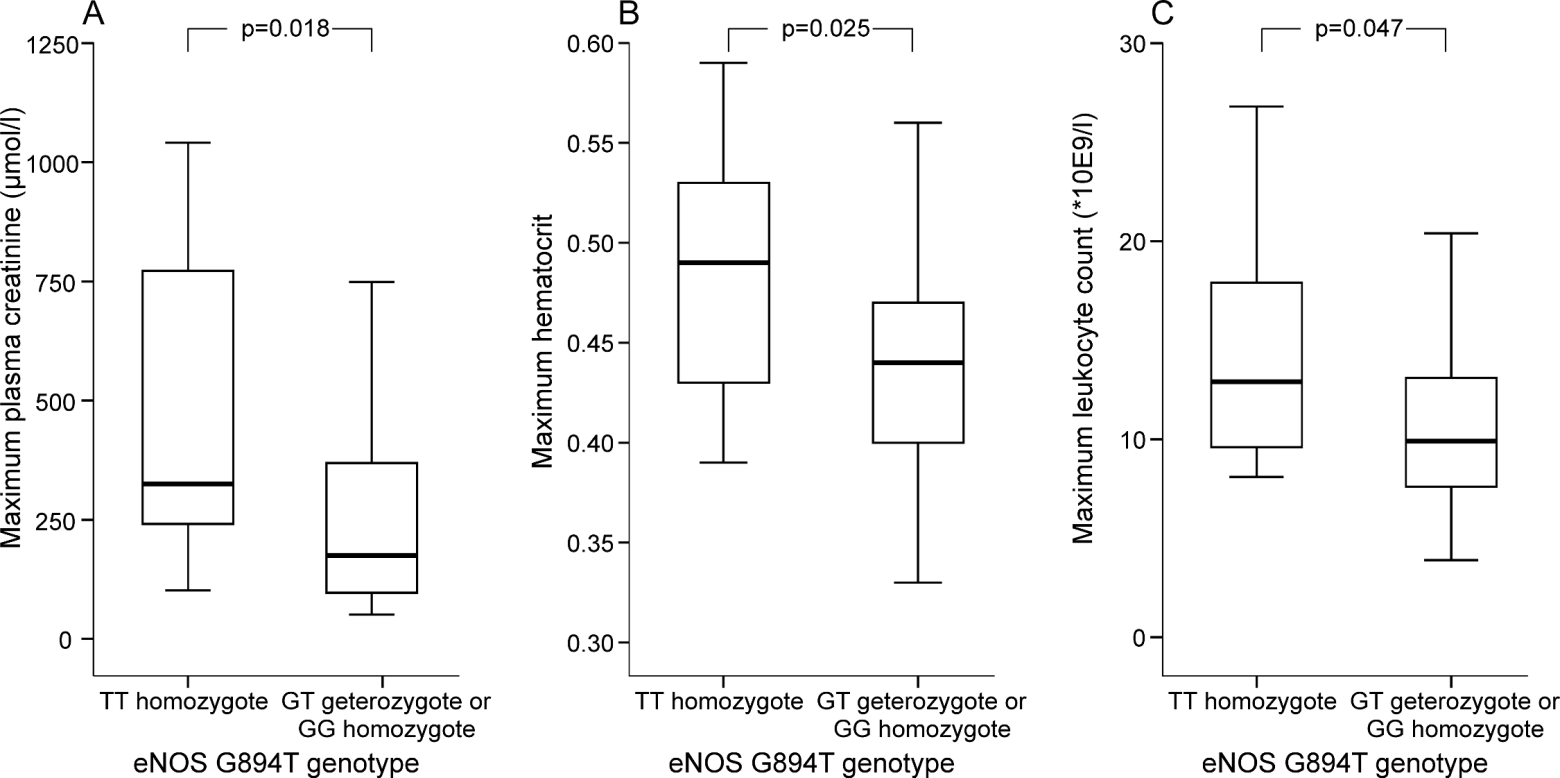
Box plots of the maximum plasma creatinine (A), hematocrit (B) and leukocyte count in TT-homozygotes (n = 10) and GT-heterozygotes or GG-homozygotes (n = 157) of eNOS G894T(rs1799983) polymorphism in 169 patients with PUUV infection. Box plot illustrates median (thick line inside box), 25^th^ and 75^th^ percentiles (box), and range (whiskers). Extremes and outliers have been omitted from the figure.

The genotype distributions and allele frequencies of eNOS G894T and iNOS G2087A polymorphisms are presented in Table 2. The rare genotype of eNOS G894T gene polymorphism was associated with the severity of AKI. The non-carriers of the G-allele of this eNOS polymorphism (TT-homozygotes, n = 10) had 1.9 times greater maximum level of serum creatinine than the carriers of the common G-allele (median 326, range 102–1041 vs. median 175, range 51–1499 μmol/l; p = 0.018, respectively: Fig 1A). The TT-homozygotes had numerically highest maximum creatinine level of all eNOS G894T genotypes, followed by the GT-heterozygotes and the GG-homozygotes, (median concentrations 326, 196, and 166 μmol/l, respectively, p = 0.061). Three out seven (43%) patients that needed hemodialysis treatment were T-allele carriers, and one of them was a TT-homozygote.

**Table 2:** The genotype distributions and allele frequencies in 167 patients for eNOS G894T (rs1799983) and in 166 patients for iNOS G2087A (rs2297518) out of 172 patients with acute Puumala hantavirus infection^*^.

	Genotype %			Allele %	
Polymorphism	homozygote common	heterozygote	homozygote rare	common	rare
eNOS(rs1799983)	59 (GG)	35 (GT)	6 (TT)	76 (G)	24 (T)
iNOS(rs2297518)	65 (GG)	33 (GA)	2 (AA)	81 (G)	19 (A)

Abbreviations: eNOS = endothelial nitride oxide synthase, iNOS = inducible nitride oxide synthase.

*The genotyping was successful in 167 patients for eNOS and in 166 patients for iNOS.

The non-carriers of the G-allele had higher maximum blood hematocrit value than the G-allele carriers (median 0.49, range 0.39–0.59 vs. median 0.44, range 0.33–0.60; p = 0.025, respectively; Fig 1B). The non-carriers of the G-allele had also higher maximum blood leukocyte count during acute phase of PUUV infection than the carriers of the common G-allele (GT-heterozygotes and GG-homozygotes, median 12.9, range 8.1–26.8 vs. median 9.9, range 3.9–31.2 *10^9^/l; p = 0.047; Fig 1C).

The length of hospital stay was longer in the non-carriers of the G-allele (TT-homozygotes) when compared with the G-allele carriers (median 8 days, range 3–14 days vs. median 6 days, range 2–15 days; p = 0.032). There were no statistically significant associations with the lowest and highest blood pressure, change in weight, lowest platelet count, maximum C-reactive protein (CRP), maximum plasma IL-6, or the need of hemodialysis treatment, and the polymorphism of eNOS G894T (S1 and S2 Files).

#### Association of iNOS G2087A (Ser608Leu, rs2297518) polymorphism with clinical and laboratory findings

Carriers of the rare A-allele of the iNOS G2087A gene (AA-homozygotes and GA-heterozygotes, n = 59) had lower minimum systolic blood pressure during PUUV infection when compared with the non-carriers (GG-homozygotes, median 110, range 74–170 vs. 116, range 86–162 mmHg, respectively; p = 0.019). Furthermore, the A-allele carriers had also lower minimum diastolic blood pressure level than the non-carriers A-allele (median 68, range 40–90 vs. 72, range 48–100 mmHg, respectively; p = 0.003).There were no significant associations with change in weight, the lowest platelet count, maximum CRP, maximum plasma IL-6, or the need of hemodialysis treatment, and the iNOS polymorphism studied here (S1 and S3 Files).

## Discussion

In this study we found that the TT-genotype of eNOS G894T polymorphism was associated with the severity of PUUV infection. PUUV-infected patients with the TT-homozygous genotype were prone to more severe AKI and longer hospital stay than the GT-heterozygotes or GG-homozygotes. They had also higher maximum leukocyte count and hematocrit values measured in the acute phase of the infection when compared with the other genotypes. The G894T polymorphism of eNOS gene was not associated with the depth of thrombocytopenia, which is in concordance with our previous finding that the severity of AKI does not associate with thrombocytopenia in acute PUUV infection in Finnish patients [33]. In some German studies severe thrombocytopenia has predicted severe AKI in PUUV infection [34, 35, 36]. We don’t know the reason for these divergent results, but genetic factors in different populations may influence.

Furthermore, we also found that the iNOS G2087A gene polymorphism was associated with decreased blood pressure. The carriers of the rare A-allele of iNOS G2087A gene variant were the most susceptible ones to suffer from severe hypotension during the acute phase of infection. However, this iNOS polymorphism was not associated with the other clinical and laboratory markers reflecting disease severity.

NO is important in maintaining vascular homeostasis via relaxation of vascular smooth muscle cells and inhibition of growth, platelet activation and aggregation, as well as leukocyte adhesion to the endothelium [37]. NO is synthesized via a calcium-dependent process in endothelial cells from the amino acid L-arginine by the constitutively expressed eNOS, *i*.*e*. NOS3 [38]. The calcium-independent formation of NO via iNOS in macrophages is mainly expressed during inflammation and infection, and is triggered by cytokines [39]. In addition to maintaining vasodilatation of the vasculature and thus controlling blood pressure, eNOS has numerous vasoprotective and anti-atherosclerotic effects [40]. Polymorphism of the NOS3 gene, localized in 7q36 region of chromosome 7 [41], seems to have functional significance. The eNOS polymorphism G894T (Glu298Asp) results in a substitution of glutamate for aspartate at position 298 in eNOS exon 7, and this change has been associated with reduced basal NO production in the forearm of healthy subjects [42].

Increased capillary permeability and vascular leakage explain many clinical features of hantavirus infection, such as hemoconcentration, hypotension, shock and tissue edema. Hantaviruses target the endothelial cells in the small vasculature [1, 9]. The data so far does not suggest that the infection would have direct cellular cytotoxicity to the endothelium, but virus-induced inflammation and host immune responses may contribute to the loss of endothelial barrier function [7, 43]. In this study, the rare G894T TT-homozygous genotype was associated with many of the clinical markers of severe PUUV infection, including hemoconcentration, leukocytosis, longer length of hospital stay, and especially the severity of AKI. As subjects with the common GG-genotype of eNOS G894T polymorphism had the mildest form of acute illness, the presence of this common G-allele might have some protective role during acute PUUV infection.

Effective renal blood flow is decreased in PUUV-induced AKI, but hypotension does not seem to explain the underlying intrarenal functional changes. Renal failure can occur without hypotension, and blood pressure levels do not correlate with the severity of AKI [9]. Renal tubular cells and mesangial cells produce NO, which is a significant regulator and also a protector of renal blood flow, glomerular filtration rate, and tubular function [44]. Interestingly, the increase in glomerular filtration rate and renal plasma flow in response to exogenous L-arginine infusion has been found to be blunted in subjects with the G894T allele of endothelial NOS, suggesting that this polymorphism is a functional variant also in human kidneys [45]. Thus, diminished NO bioavailability due to eNOS G894T polymorphism could predispose to the impairment of vascular and renal function through vasoconstriction.

Polymorphism of iNOS G2087A (Ser608Leu) leads to an amino acid substitution from serine to leucine in the coding region of exon 16 in NOS2 [30]. This gene variant is supposed to promote excessive NO formation and inflammation through increased iNOS activity within the A-allele carriers. In macrophages NO is a mediator of tumoricidal and bactericidal actions [30]. Previous studies have indicated that iNOS plays an important role in the origin of hypotension in septic shock. The A-allele carriage has been associated with increased susceptibility to septic shock [29]. Our finding indicated that the rare A-allele carriers (*i*.*e*. GA-heterozygotes and AA-homozygotes) of iNOS G2087A gene variant also suffered from more severe hypotension than the non-carriers of A-allele during acute PUUV infection.

We recently reported two cases of PUUV-HFRS with severe capillary leakage syndrome that were successfully treated with icatibant, a bradykinin B2-receptor antagonist [46, 47]. The activation of the kinin-kallikrein system and the subsequent formation of bradykinin is enhanced in hantavirus-infected endothelial cells [48]. The synthesis of eNOS is activated by bradykinin, which causes blood vessels to dilate via the release of NO and other endothelial autacoids [49]. Interestingly, it has been demonstrated that there is an association between HFRS and acute myocardial infarction and stroke in the acute phase of the disease, which may be partly explained by the increased platelet activation [50, 51]. Many studies have implicated eNOS polymorphism in the development of cardiovascular diseases [52], and the homozygous mutant (TT) genotype of G894T has conferred increased susceptibility to CAD [53, 54]. In the present study, the TT-homozygotes had the most severe AKI as evaluated by maximum creatinine levels, followed by the GT-heterozygotes and the GG-homozygotes. Although the number of the TT-homozygotes in our study here was only 10 patients, the results well correspond to the other findings above that have been associated with the G894T polymorphism of the eNOS gene. Taken together, our findings point to the possibility of impaired constitutive NO synthesis in the pathogenesis of acute hantavirus infection.

In conclusion, this study implies that eNOS G894T polymorphism may influence the clinical course of PUUV infection. This eNOS gene variant, associated with various vascular diseases, may also play some part in the endothelial and kidney dysfunction in the complex pathogenesis of acute PUUV infection. Among PUUV-infected patients, those with the rare TT-genotype of eNOS G894T polymorphism were more susceptible to severe AKI. Moreover, patients with the rare A-allele of iNOS G2087A polymorphism had more severe hypotension during the acute phase of infection. To our knowledge this is the first study to associate eNOS and iNOS polymorphisms and disease severity of HFRS.

## Supporting Information

S1 File ENOS G894T polymorphism and associations with clinical and laboratory variables in PUUV infection. (PDF)

S2 File ENOS G894T polymorphism and associations with clinical and laboratory variables in PUUV infection. (PDF)

S3 File INOS G2087A polymorphism and associations with clinical and laboratory variables in PUUV infection. (PDF)
