# Supplementary material for: Endothelial Nitric Oxide Synthase G894T Polymorphism Associates with Disease Severity in Puumala Hantavirus Infection
Source: PLoS One. 2015 Nov 11;10(11):e0142872. doi: 10.1371/journal.pone.0142872 (PMC4641644; doi:10.1371/journal.pone.0142872)
Supplement: S1 File — (PDF) [file pone.0142872.s001.pdf]

```
GET
  FILE='C:\Users\mehehu\AppData\Local\Microsoft\Windows\Temporary Internet Files\Content.Outlook\ELK0KF
DATASET NAME DataSet1 WINDOW=FRONT.

SAVE OUTFILE='D:\mehehu\mehehu_datat_C\tutkimus\projektit\Outi_l\Outi_K\polymorfiat.sav'
  /COMPRESSED.
FREQUENCIES VARIABLES=enosnum inosnum estrognum
  /ORDER=ANALYSIS.
```

1) Enos-, inos- ja estr1- genotyyppien (variaabelitenosnum, inosnumjaestrognum) ja enos-, inos- ja estr-kantajuuksien (carAinos, carGinos, carGenos, carTenos, carTestr ja carCestr) p-arvot/assosiaatiot vielä seuraaviin kliinisiin muuttujiin:oloaika, sbpmax, sbpmin, dbpmin, dbpmax, dialyysi

[DataSet1] D:\mehehu\mehehu_datat_C\tutkimus\projektit\Outi_l\Outi_K\polymorfiat.sav

Statistics

		enosnum	inosnum	estrogeenireseptori numeerisesti
N	Valid	172	172	172
	Missing	0	0	0

Frequency Table

enosnum

		Frequency	Percent	Valid Percent	Cumulative Percent
Valid	G	98	57.0	57.0	57.0
	Both	59	34.3	34.3	91.3
	T	10	5.8	5.8	97.1
	Undeterm	5	2.9	2.9	100.0
	Total	172	100.0	100.0	

inosnum

		Frequency	Percent	Valid Percent	Cumulative Percent
Valid	G	107	62.2	62.2	62.2
	Both	55	32.0	32.0	94.2
	A	4	2.3	2.3	96.5
	Undeterm	6	3.5	3.5	100.0
	Total	172	100.0	100.0	

estrogeenireseptori numeerisesti

		Frequency	Percent	Valid Percent	Cumulative Percent
Valid	T	52	30.2	30.2	30.2
	Both	82	47.7	47.7	77.9
	C	29	16.9	16.9	94.8
	Undeter	9	5.2	5.2	100.0
	Total	172	100.0	100.0	

```
GET
  FILE='D:\mehehu\mehehu_datat_C\tutkimus\projektit\Outi_l\Outi_K\polymorfiat2.sav'.
DATASET NAME DataSet2 WINDOW=FRONT.
```

```
DATASET ACTIVATE DataSet1.
FREQUENCIES VARIABLES=carAinos carGinos carGenos carTenos carTestr carCestr
  /ORDER=ANALYSIS.
```

Frequencies

[DataSet1] D:\mehehu\mehehu_datat_C\tutkimus\projektit\Outi_l\Outi_K\polymorfiat.sav

Statistics

		inos A-alleelin kantaja	inos G-alleelin kantaja	enos G-alleelin kantaja	enos T-alleelin kantaja	estr T-alleelin kantaja	estr C-alleelin kantaja
N	Valid	166	166	167	167	163	163
	Missing	6	6	5	5	9	9

Frequency Table

inos A-alleelin kantaja

		Frequency	Percent	Valid Percent	Cumulative Percent
Valid	ei kanna A-alleelia	107	62.2	64.5	64.5
	kantaa A-alleelia	59	34.3	35.5	100.0
	Total	166	96.5	100.0	
Missing	System	6	3.5		
Total		172	100.0		

inos G-alleelin kantaja

		Frequency	Percent	Valid Percent	Cumulative Percent
Valid	ei kanna G alleelia	4	2.3	2.4	2.4
	kantaa G-alleelia	162	94.2	97.6	100.0
	Total	166	96.5	100.0	
Missing	System	6	3.5		
Total		172	100.0		

enos G-alleelin kantaja

		Frequency	Percent	Valid Percent	Cumulative Percent
Valid	ei kanna G-alleelia	10	5.8	6.0	6.0
	kantaa G-alleelia	157	91.3	94.0	100.0
	Total	167	97.1	100.0	
Missing	System	5	2.9		
Total		172	100.0		

enos T-alleelin kantaja

		Frequency	Percent	Valid Percent	Cumulative Percent
Valid	ei kanna T-alleelia	98	57.0	58.7	58.7
	kantaa T-alleelia	69	40.1	41.3	100.0
	Total	167	97.1	100.0	
Missing	System	5	2.9		
Total		172	100.0		

estr T-alleelin kantaja

		Frequency	Percent	Valid Percent	Cumulative Percent
Valid	ei kanna T-alleelia	29	16.9	17.8	17.8
	kantaa T-alleelia	134	77.9	82.2	100.0
	Total	163	94.8	100.0	
Missing	System	9	5.2		
Total		172	100.0		

estr C-alleelin kantaja

		Frequency	Percent	Valid Percent	Cumulative Percent
Valid	ei ole alleelin kantaja	52	30.2	31.9	31.9
	alleelin kantaja	111	64.5	68.1	100.0
	Total	163	94.8	100.0	
Missing	System	9	5.2		
Total		172	100.0		

```
DATASET ACTIVATE DataSet2.
SORT CASES  BY enosnum.
SPLIT FILE LAYERED BY enosnum.


FREQUENCIES VARIABLES=oloaika sbpmax sbpmin dbpmin dbpmax
  /FORMAT=NOTABLE
  /NTILES=4
  /STATISTICS=STDDEV MINIMUM MAXIMUM MEAN MEDIAN
  /ORDER=ANALYSIS.
```

Frequencies

```
[DataSet2] D:\mehehu\mehehu_datat_C\tutkimus\projektit\Outi_l\Outi_K\polymorfiat2.sav
```

Statistics

enosnum			oloaika	sbpmax	sbpmin	dbpmin	dbpmax
G	N	Valid	98	97	97	97	97
		Missing	0	1	1	1	1
	Mean		6.3878	139.5670	113.5773	70.0722	86.9897
	Median		6.0000	135.0000	111.0000	70.0000	86.0000
	Std. Deviation		2.30925	19.78210	15.47702	9.55603	10.69316
	Minimum		3.00	102.00	85.00	45.00	64.00
	Maximum		15.00	210.00	170.00	92.00	112.00
	Percentiles	25	5.0000	126.5000	104.0000	62.0000	80.0000
		50	6.0000	135.0000	111.0000	70.0000	86.0000
		75	8.0000	148.0000	121.5000	77.5000	94.5000
Both	N	Valid	59	59	59	58	59
		Missing	0	0	0	1	0
	Mean		6.4237	141.3729	115.8136	70.6379	89.2881
	Median		6.0000	141.0000	115.0000	70.0000	89.0000
	Std. Deviation		2.67945	19.19581	16.96088	10.11287	12.81305
	Minimum		2.00	107.00	74.00	40.00	67.00
	Maximum		15.00	204.00	155.00	91.00	120.00
	Percentiles	25	5.0000	126.0000	106.0000	65.7500	80.0000
		50	6.0000	141.0000	115.0000	70.0000	89.0000
		75	8.0000	154.0000	126.0000	79.0000	98.0000
T	N	Valid	10	10	10	10	10
		Missing	0	0	0	0	0
	Mean		8.2000	148.8000	116.1000	67.2000	93.1000
	Median		8.0000	147.5000	111.0000	64.5000	93.0000
	Std. Deviation		2.93636	28.78580	24.01134	13.77437	16.57609
	Minimum		3.00	95.00	86.00	48.00	60.00
	Maximum		14.00	192.00	162.00	100.00	110.00
	Percentiles	25	6.7500	132.0000	98.0000	60.0000	82.0000
		50	8.0000	147.5000	111.0000	64.5000	93.0000
		75	10.0000	169.7500	128.7500	71.7500	109.2500
Undeterm	N	Valid	5	5	5	5	5
		Missing	0	0	0	0	0
	Mean		5.4000	125.2000	109.2000	65.6000	76.8000
	Median		4.0000	120.0000	110.0000	65.0000	72.0000
	Std. Deviation		2.60768	18.45806	9.83362	6.87750	10.75639
	Minimum		4.00	106.00	94.00	58.00	69.00
	Maximum		10.00	154.00	120.00	74.00	95.00
	Percentiles	25	4.0000	110.5000	100.5000	59.0000	69.5000
		50	4.0000	120.0000	110.0000	65.0000	72.0000
		75	7.5000	142.5000	117.5000	72.5000	86.5000

```
SPLIT FILE OFF.
NPAR TESTS
  /K-W=oloaika sbpmax sbpmin dbpmin dbpmax BY enosnum(1 3)
  /MISSING ANALYSIS.
```

NPar Tests

```
[DataSet2] D:\mehehu\mehehu_datat_C\tutkimus\projektit\Outi_l\Outi_K\polymorfiat2.sav
```

Kruskal-Wallis Test

Ranks

	enosnum	N	Mean Rank
oloaika	G	98	82.23
	Both	59	81.59
	T	10	115.55
	Total	167	
sbpmax	G	97	79.38
	Both	59	86.81
	T	10	104.00
	Total	166	
sbpmin	G	97	79.94
	Both	59	90.08
	T	10	79.15
	Total	166	
dbpmin	G	97	82.25
	Both	58	87.85
	T	10	62.15
	Total	165	
dbpmax	G	97	79.23
	Both	59	87.02
	T	10	104.15
	Total	166	

Test Statistics[a,b]

	oloaika	sbpmax	sbpmin	dbpmin	dbpmax
Chi-Square	4.614	2.814	1.722	2.533	2.930
df	2	2	2	2	2
Asymp. Sig.	.100	.245	.423	.282	.231

a. Kruskal Wallis Test

b. Grouping Variable: enosnum

```
*** inosnum ***.
SORT CASES  BY inosnum.
SPLIT FILE LAYERED BY inosnum.


FREQUENCIES VARIABLES=oloaika sbpmax sbpmin dbpmin dbpmax
  /FORMAT=NOTABLE
  /NTILES=4
  /STATISTICS=STDDEV MINIMUM MAXIMUM MEAN MEDIAN
  /ORDER=ANALYSIS.
```

Frequencies

```
[DataSet2] D:\mehehu\mehehu_datat_C\tutkimus\projektit\Outi_l\Outi_K\polymorfiat2.sav
```

Statistics

inosnum			oloaika	sbpmax	sbpmin	dbpmin	dbpmax
G	N	Valid	107	106	106	106	106
		Missing	0	1	1	1	1
	Mean		6.3738	142.6226	117.0377	72.0000	89.0755
	Median		6.0000	140.0000	115.5000	71.5000	89.0000
	Std. Deviation		2.33724	20.65381	15.98864	9.14851	12.36331
	Minimum		2.00	95.00	86.00	48.00	60.00
	Maximum		14.00	210.00	162.00	100.00	120.00
	Percentiles	25	5.0000	130.0000	106.7500	66.0000	80.0000
		50	6.0000	140.0000	115.5000	71.5000	89.0000
		75	8.0000	156.2500	125.0000	78.2500	98.2500
Both	N	Valid	55	55	55	54	55
		Missing	0	0	0	1	0
	Mean		6.9091	138.5455	111.2182	66.7963	87.3273
	Median		7.0000	134.0000	110.0000	66.5000	87.0000
	Std. Deviation		2.90129	19.47060	16.66855	10.46897	10.94101
	Minimum		3.00	111.00	74.00	40.00	64.00
	Maximum		15.00	198.00	170.00	90.00	109.00
	Percentiles	25	5.0000	125.0000	100.0000	60.0000	80.0000
		50	7.0000	134.0000	110.0000	66.5000	87.0000
		75	8.0000	147.0000	120.0000	74.0000	94.0000
A	N	Valid	4	4	4	4	4
		Missing	0	0	0	0	0
	Mean		5.0000	130.0000	111.5000	69.2500	77.5000
	Median		5.0000	131.0000	111.5000	71.0000	78.5000
	Std. Deviation		.81650	6.68331	2.38048	6.50000	7.59386
	Minimum		4.00	122.00	109.00	60.00	68.00
	Maximum		6.00	136.00	114.00	75.00	85.00
	Percentiles	25	4.2500	123.2500	109.2500	62.5000	69.7500
		50	5.0000	131.0000	111.5000	71.0000	78.5000
		75	5.7500	135.7500	113.7500	74.2500	84.2500
Undeterm	N	Valid	6	6	6	6	6
		Missing	0	0	0	0	0
	Mean		5.3333	122.5000	98.0000	63.0000	77.6667
	Median		5.0000	126.0000	94.5000	62.5000	75.0000
	Std. Deviation		1.50555	15.99687	12.64911	11.78134	10.98484
	Minimum		4.00	102.00	85.00	45.00	66.00
	Maximum		8.00	142.00	115.00	79.00	96.00
	Percentiles	25	4.0000	105.0000	86.5000	54.7500	69.0000
		50	5.0000	126.0000	94.5000	62.5000	75.0000
		75	6.5000	135.2500	112.7500	73.0000	87.0000

```
SPLIT FILE OFF.
NPAR TESTS
  /K-W=oloaika sbpmax sbpmin dbpmin dbpmax BY inosnum(1 3)
  /MISSING ANALYSIS.
```

NPar Tests

[DataSet2] D:\mehehu\mehehu_datat_C\tutkimus\projektit\Outi_l\Outi_K\polymorfiat2.sav

Kruskal-Wallis Test

Ranks

	inosnum	N	Mean Rank
oloaika	G	107	82.10
	Both	55	88.50
	A	4	52.25
	Total	166	
sbpmax	G	106	88.21
	Both	55	75.03
	A	4	54.63
	Total	165	
sbpmin	G	106	89.52
	Both	55	71.25
	A	4	71.75
	Total	165	
dbpmin	G	106	90.60
	Both	54	66.90
	A	4	78.38
	Total	164	
dbpmax	G	106	85.78
	Both	55	80.80
	A	4	39.50
	Total	165	

Test Statistics[a,b]

	oloaika	sbpmax	sbpmin	dbpmin	dbpmax
Chi-Square	2.417	4.205	5.534	8.969	3.797
df	2	2	2	2	2
Asymp. Sig.	.299	.122	.063	.011	.150

a. Kruskal Wallis Test

b. Grouping Variable: inosnum

```
*** estrognum ***.
SORT CASES  BY estrognum.
SPLIT FILE LAYERED BY estrognum.


FREQUENCIES VARIABLES=oloaika sbpmax sbpmin dbpmin dbpmax
  /FORMAT=NOTABLE
  /NTILES=4
  /STATISTICS=STDDEV MINIMUM MAXIMUM MEAN MEDIAN
  /ORDER=ANALYSIS.
```

Frequencies

```
[DataSet2] D:\mehehu\mehehu_datat_C\tutkimus\projektit\Outi_l\Outi_K\polymorfiat2.sav
```

Statistics

estrogeenireseptori numeerisesti			oloaika	sbpmax	sbpmin	dbpmin	dbpmax
T	N	Valid	52	51	51	50	51
		Missing	0	1	1	2	1
	Mean		6.5000	139.8431	113.4902	71.1800	87.3529
	Median		6.0000	136.0000	112.0000	72.0000	88.0000
	Std. Deviation		2.85945	18.86730	14.87733	8.40236	11.15854
	Minimum		2.00	107.00	86.00	54.00	67.00
	Maximum		15.00	188.00	149.00	90.00	113.00
	Percentiles	25	4.2500	125.0000	104.0000	64.2500	78.0000
		50	6.0000	136.0000	112.0000	72.0000	88.0000
		75	8.0000	148.0000	120.0000	79.0000	96.0000
Both	N	Valid	82	82	82	82	82
		Missing	0	0	0	0	0
	Mean		6.6829	140.4146	115.1098	70.2317	88.2317
	Median		6.0000	137.5000	113.0000	70.0000	88.0000
	Std. Deviation		2.47395	19.88234	15.73861	9.86989	12.17090
	Minimum		3.00	95.00	82.00	40.00	60.00
	Maximum		15.00	210.00	162.00	100.00	112.00
	Percentiles	25	5.0000	127.2500	105.0000	64.0000	80.0000
		50	6.0000	137.5000	113.0000	70.0000	88.0000
		75	9.0000	150.7500	124.0000	78.0000	95.2500
C	N	Valid	29	29	29	29	29
		Missing	0	0	0	0	0
	Mean		6.2414	143.1379	113.3103	66.7931	89.7241
	Median		7.0000	139.0000	113.0000	68.0000	88.0000
	Std. Deviation		2.06424	23.58075	20.46793	11.99815	12.85606
	Minimum		2.00	102.00	74.00	40.00	66.00
	Maximum		10.00	204.00	170.00	91.00	120.00
	Percentiles	25	4.0000	129.0000	99.5000	60.0000	83.0000
		50	7.0000	139.0000	113.0000	68.0000	88.0000
		75	7.0000	156.0000	124.5000	73.0000	100.0000
Undeter	N	Valid	9	9	9	9	9
		Missing	0	0	0	0	0
	Mean		5.2222	132.8889	116.0000	71.0000	81.0000
	Median		5.0000	130.0000	115.0000	71.0000	78.0000
	Std. Deviation		1.71594	21.25702	18.03469	10.78193	11.80042
	Minimum		3.00	106.00	94.00	58.00	65.00
	Maximum		8.00	173.00	157.00	92.00	105.00
	Percentiles	25	3.5000	118.5000	103.5000	62.0000	72.5000
		50	5.0000	130.0000	115.0000	71.0000	78.0000
		75	6.5000	147.0000	119.5000	77.5000	88.0000

```
SPLIT FILE OFF.
NPAR TESTS
  /K-W=oloaika sbpmax sbpmin dbpmin dbpmax BY estrognum(1 3)
  /MISSING ANALYSIS.
```

NPar Tests

[DataSet2] D:\mehehu\mehehu_datat_C\tutkimus\projektit\Outi_l\Outi_K\polymorfiat2.sav

Kruskal-Wallis Test

Ranks

	estrogeenireseptori numeerisesti	N	Mean Rank
oloaika	T	52	79.78
	Both	82	84.30
	C	29	79.48
	Total	163	
sbpmax	T	51	80.03
	Both	82	81.20
	C	29	84.95
	Total	162	
sbpmin	T	51	79.94
	Both	82	83.51
	C	29	78.55
	Total	162	
dbpmin	T	50	87.74
	Both	82	81.99
	C	29	66.57
	Total	161	
dbpmax	T	51	77.93
	Both	82	81.76
	C	29	87.05
	Total	162	

Test Statistics[a,b]

	oloaika	sbpmax	sbpmin	dbpmin	dbpmax
Chi-Square	.399	.210	.322	3.871	.705
df	2	2	2	2	2
Asymp. Sig.	.819	.900	.851	.144	.703

a. Kruskal Wallis Test

b. Grouping Variable: estrogeenireseptori numeerisesti

```
DATASET ACTIVATE DataSet1.
****** carAinos.***.
SORT CASES  BY carAinos.
SPLIT FILE LAYERED BY carAinos.


FREQUENCIES VARIABLES=oloaika sbpmax sbpmin dbpmin dbpmax
  /FORMAT=NOTABLE
  /NTILES=4
  /STATISTICS=STDDEV MINIMUM MAXIMUM MEAN MEDIAN
  /ORDER=ANALYSIS.
```

Frequencies

```
[DataSet1] D:\mehehu\mehehu_datat_C\tutkimus\projektit\Outi_l\Outi_K\polymorfiat.sav
```

Statistics

inos A-alleelin kantaja			oloaika	sbpmax	sbpmin	dbpmin	dbpmax
.00	N	Valid	6	6	6	6	6
		Missing	0	0	0	0	0
	Mean		5.3333	122.5000	98.0000	63.0000	77.6667
	Median		5.0000	126.0000	94.5000	62.5000	75.0000
	Std. Deviation		1.50555	15.99687	12.64911	11.78134	10.98484
	Minimum		4.00	102.00	85.00	45.00	66.00
	Maximum		8.00	142.00	115.00	79.00	96.00
	Percentiles	25	4.0000	105.0000	86.5000	54.7500	69.0000
		50	5.0000	126.0000	94.5000	62.5000	75.0000
		75	6.5000	135.2500	112.7500	73.0000	87.0000
ei kanna A-alleelia	N	Valid	107	106	106	106	106
		Missing	0	1	1	1	1
	Mean		6.3738	142.6226	117.0377	72.0000	89.0755
	Median		6.0000	140.0000	115.5000	71.5000	89.0000
	Std. Deviation		2.33724	20.65381	15.98864	9.14851	12.36331
	Minimum		2.00	95.00	86.00	48.00	60.00
	Maximum		14.00	210.00	162.00	100.00	120.00
	Percentiles	25	5.0000	130.0000	106.7500	66.0000	80.0000
		50	6.0000	140.0000	115.5000	71.5000	89.0000
		75	8.0000	156.2500	125.0000	78.2500	98.2500
kantaa A-alleelia	N	Valid	59	59	59	58	59
		Missing	0	0	0	1	0
	Mean		6.7797	137.9661	111.2373	66.9655	86.6610
	Median		6.0000	134.0000	110.0000	68.0000	86.0000
	Std. Deviation		2.84707	18.97273	16.09277	10.22375	10.98370
	Minimum		3.00	111.00	74.00	40.00	64.00
	Maximum		15.00	198.00	170.00	90.00	109.00
	Percentiles	25	5.0000	125.0000	102.0000	60.0000	80.0000
		50	6.0000	134.0000	110.0000	68.0000	86.0000
		75	8.0000	143.0000	119.0000	74.0000	94.0000

```
SPLIT FILE OFF.
NPAR TESTS
  /M-W= oloaika sbpmax sbpmin dbpmin dbpmax BY carAinos(0 1)
  /MISSING ANALYSIS.
```

NPar Tests

```
[DataSet1] D:\mehehu\mehehu_datat_C\tutkimus\projektit\Outi_l\Outi_K\polymorfiat.sav
```

Mann-Whitney Test

Ranks

	inos A-alleelin kantaja	N	Mean Rank	Sum of Ranks
oloaika	ei kanna A-alleelia	107	82.10	8784.50
	kantaa A-alleelia	59	86.04	5076.50
	Total	166		
sbpmax	ei kanna A-alleelia	106	88.21	9350.00
	kantaa A-alleelia	59	73.64	4345.00
	Total	165		
sbpmin	ei kanna A-alleelia	106	89.52	9489.50
	kantaa A-alleelia	59	71.28	4205.50
	Total	165		
dbpmin	ei kanna A-alleelia	106	90.60	9604.00
	kantaa A-alleelia	58	67.69	3926.00
	Total	164		
dbpmax	ei kanna A-alleelia	106	85.78	9093.00
	kantaa A-alleelia	59	78.00	4602.00
	Total	165		

Test Statistics[a]

	oloaika	sbpmax	sbpmin	dbpmin	dbpmax
Mann-Whitney U	3006.500	2575.000	2435.500	2215.000	2832.000
Wilcoxon W	8784.500	4345.000	4205.500	3926.000	4602.000
Z	-.510	-1.877	-2.352	-2.958	-1.004
Asymp. Sig. (2-tailed)	.610	.060	.019	.003	.316

a. Grouping Variable: inos A-alleelin kantaja

```
****** carGinos.***.
SORT CASES  BY carGinos.
SPLIT FILE LAYERED BY carGinos.


FREQUENCIES VARIABLES=oloaika sbpmax sbpmin dbpmin dbpmax
  /FORMAT=NOTABLE
  /NTILES=4
  /STATISTICS=STDDEV MINIMUM MAXIMUM MEAN MEDIAN
  /ORDER=ANALYSIS.
```

Frequencies

```
[DataSet1] D:\mehehu\mehehu_datat_C\tutkimus\projektit\Outi_l\Outi_K\polymorfiat.sav
```

Statistics

inos G-alleelin kantaja			oloaika	sbpmax	sbpmin	dbpmin	dbpmax
.00	N	Valid	6	6	6	6	6
		Missing	0	0	0	0	0
	Mean		5.3333	122.5000	98.0000	63.0000	77.6667
	Median		5.0000	126.0000	94.5000	62.5000	75.0000
	Std. Deviation		1.50555	15.99687	12.64911	11.78134	10.98484
	Minimum		4.00	102.00	85.00	45.00	66.00
	Maximum		8.00	142.00	115.00	79.00	96.00
	Percentiles	25	4.0000	105.0000	86.5000	54.7500	69.0000
		50	5.0000	126.0000	94.5000	62.5000	75.0000
		75	6.5000	135.2500	112.7500	73.0000	87.0000
ei kanna G alleelia	N	Valid	4	4	4	4	4
		Missing	0	0	0	0	0
	Mean		5.0000	130.0000	111.5000	69.2500	77.5000
	Median		5.0000	131.0000	111.5000	71.0000	78.5000
	Std. Deviation		.81650	6.68331	2.38048	6.50000	7.59386
	Minimum		4.00	122.00	109.00	60.00	68.00
	Maximum		6.00	136.00	114.00	75.00	85.00
	Percentiles	25	4.2500	123.2500	109.2500	62.5000	69.7500
		50	5.0000	131.0000	111.5000	71.0000	78.5000
		75	5.7500	135.7500	113.7500	74.2500	84.2500
kantaa G-alleelia	N	Valid	162	161	161	160	161
		Missing	0	1	1	2	1
	Mean		6.5556	141.2298	115.0497	70.2438	88.4783
	Median		6.0000	139.0000	114.0000	70.0000	88.0000
	Std. Deviation		2.54646	20.28924	16.40724	9.89425	11.89122
	Minimum		2.00	95.00	74.00	40.00	60.00
	Maximum		15.00	210.00	170.00	100.00	120.00
	Percentiles	25	5.0000	126.0000	104.5000	62.2500	80.0000
		50	6.0000	139.0000	114.0000	70.0000	88.0000
		75	8.0000	154.0000	124.0000	77.7500	96.0000

```
SPLIT FILE OFF.
NPAR TESTS
  /M-W= oloaika sbpmax sbpmin dbpmin dbpmax BY carGinos(0 1)
  /MISSING ANALYSIS.
```

NPar Tests

```
[DataSet1] D:\mehehu\mehehu_datat_C\tutkimus\projektit\Outi_l\Outi_K\polymorfiat.sav
```

Mann-Whitney Test

Ranks

	inos G-alleelin kantaja	N	Mean Rank	Sum of Ranks
oloaika	ei kanna G alleelia	4	52.25	209.00
	kantaa G-alleelia	162	84.27	13652.00
	Total	166		
sbpmax	ei kanna G alleelia	4	54.63	218.50
	kantaa G-alleelia	161	83.70	13476.50
	Total	165		
sbpmin	ei kanna G alleelia	4	71.75	287.00
	kantaa G-alleelia	161	83.28	13408.00
	Total	165		
dbpmin	ei kanna G alleelia	4	78.38	313.50
	kantaa G-alleelia	160	82.60	13216.50
	Total	164		
dbpmax	ei kanna G alleelia	4	39.50	158.00
	kantaa G-alleelia	161	84.08	13537.00
	Total	165		

Test Statistics[a]

	oloaika	sbpmax	sbpmin	dbpmin	dbpmax
Mann-Whitney U	199.000	208.500	277.000	303.500	148.000
Wilcoxon W	209.000	218.500	287.000	313.500	158.000
Z	-1.327	-1.203	-.477	-.176	-1.845
Asymp. Sig. (2-tailed)	.184	.229	.633	.860	.065

a. Grouping Variable: inos G-alleelin kantaja

```
****** carGenos.***.
SORT CASES  BY carGenos.
SPLIT FILE LAYERED BY carGenos.


FREQUENCIES VARIABLES=oloaika sbpmax sbpmin dbpmin dbpmax
  /FORMAT=NOTABLE
  /NTILES=4
  /STATISTICS=STDDEV MINIMUM MAXIMUM MEAN MEDIAN
  /ORDER=ANALYSIS.
```

Frequencies

```
[DataSet1] D:\mehehu\mehehu_datat_C\tutkimus\projektit\Outi_l\Outi_K\polymorfiat.sav
```

Statistics

enos G-alleelin kantaja			oloaika	sbpmax	sbpmin	dbpmin	dbpmax
.00	N	Valid	5	5	5	5	5
		Missing	0	0	0	0	0
	Mean		5.4000	125.2000	109.2000	65.6000	76.8000
	Median		4.0000	120.0000	110.0000	65.0000	72.0000
	Std. Deviation		2.60768	18.45806	9.83362	6.87750	10.75639
	Minimum		4.00	106.00	94.00	58.00	69.00
	Maximum		10.00	154.00	120.00	74.00	95.00
	Percentiles	25	4.0000	110.5000	100.5000	59.0000	69.5000
		50	4.0000	120.0000	110.0000	65.0000	72.0000
		75	7.5000	142.5000	117.5000	72.5000	86.5000
ei kanna G-alleelia	N	Valid	10	10	10	10	10
		Missing	0	0	0	0	0
	Mean		8.2000	148.8000	116.1000	67.2000	93.1000
	Median		8.0000	147.5000	111.0000	64.5000	93.0000
	Std. Deviation		2.93636	28.78580	24.01134	13.77437	16.57609
	Minimum		3.00	95.00	86.00	48.00	60.00
	Maximum		14.00	192.00	162.00	100.00	110.00
	Percentiles	25	6.7500	132.0000	98.0000	60.0000	82.0000
		50	8.0000	147.5000	111.0000	64.5000	93.0000
		75	10.0000	169.7500	128.7500	71.7500	109.2500
kantaa G-alleelia	N	Valid	157	156	156	155	156
		Missing	0	1	1	2	1
	Mean		6.4013	140.2500	114.4231	70.2839	87.8590
	Median		6.0000	137.0000	113.5000	70.0000	87.5000
	Std. Deviation		2.44650	19.51992	16.03707	9.73930	11.55433
	Minimum		2.00	102.00	74.00	40.00	64.00
	Maximum		15.00	210.00	170.00	92.00	120.00
	Percentiles	25	5.0000	126.0000	104.0000	63.0000	80.0000
		50	6.0000	137.0000	113.5000	70.0000	87.5000
		75	8.0000	150.0000	123.7500	78.0000	95.7500

```
SPLIT FILE OFF.
NPAR TESTS
  /M-W= oloaika sbpmax sbpmin dbpmin dbpmax BY carGenos(0 1)
  /MISSING ANALYSIS.
```

NPar Tests

```
[DataSet1] D:\mehehu\mehehu_datat_C\tutkimus\projektit\Outi_l\Outi_K\polymorfiat.sav
```

Mann-Whitney Test

Ranks

	enos G-alleelin kantaja	N	Mean Rank	Sum of Ranks
oloaika	ei kanna G-alleelia	10	115.55	1155.50
	kantaa G-alleelia	157	81.99	12872.50
	Total	167		
sbpmax	ei kanna G-alleelia	10	104.00	1040.00
	kantaa G-alleelia	156	82.19	12821.00
	Total	166		
sbpmin	ei kanna G-alleelia	10	79.15	791.50
	kantaa G-alleelia	156	83.78	13069.50
	Total	166		
dbpmin	ei kanna G-alleelia	10	62.15	621.50
	kantaa G-alleelia	155	84.35	13073.50
	Total	165		
dbpmax	ei kanna G-alleelia	10	104.15	1041.50
	kantaa G-alleelia	156	82.18	12819.50
	Total	166		

Test Statistics[a]

	oloaika	sbpmax	sbpmin	dbpmin	dbpmax
Mann-Whitney U	469.500	575.000	736.500	566.500	573.500
Wilcoxon W	12872.500	12821.000	791.500	621.500	12819.500
Z	-2.146	-1.392	-.295	-1.426	-1.402
Asymp. Sig. (2-tailed)	.032	.164	.768	.154	.161

a. Grouping Variable: enos G-alleelin kantaja

```
****** carTenos.***.
SORT CASES  BY carTenos.
SPLIT FILE LAYERED BY carTenos.


FREQUENCIES VARIABLES=oloaika sbpmax sbpmin dbpmin dbpmax
  /FORMAT=NOTABLE
  /NTILES=4
  /STATISTICS=STDDEV MINIMUM MAXIMUM MEAN MEDIAN
  /ORDER=ANALYSIS.
```

Frequencies

```
[DataSet1] D:\mehehu\mehehu_datat_C\tutkimus\projektit\Outi_l\Outi_K\polymorfiat.sav
```

Statistics

enos T-alleelin kantaja			oloaika	sbpmax	sbpmin	dbpmin	dbpmax
.00	N	Valid	5	5	5	5	5
		Missing	0	0	0	0	0
	Mean		5.4000	125.2000	109.2000	65.6000	76.8000
	Median		4.0000	120.0000	110.0000	65.0000	72.0000
	Std. Deviation		2.60768	18.45806	9.83362	6.87750	10.75639
	Minimum		4.00	106.00	94.00	58.00	69.00
	Maximum		10.00	154.00	120.00	74.00	95.00
	Percentiles	25	4.0000	110.5000	100.5000	59.0000	69.5000
		50	4.0000	120.0000	110.0000	65.0000	72.0000
		75	7.5000	142.5000	117.5000	72.5000	86.5000
ei kanna T-alleelia	N	Valid	98	97	97	97	97
		Missing	0	1	1	1	1
	Mean		6.3878	139.5670	113.5773	70.0722	86.9897
	Median		6.0000	135.0000	111.0000	70.0000	86.0000
	Std. Deviation		2.30925	19.78210	15.47702	9.55603	10.69316
	Minimum		3.00	102.00	85.00	45.00	64.00
	Maximum		15.00	210.00	170.00	92.00	112.00
	Percentiles	25	5.0000	126.5000	104.0000	62.0000	80.0000
		50	6.0000	135.0000	111.0000	70.0000	86.0000
		75	8.0000	148.0000	121.5000	77.5000	94.5000
kantaa T-alleelia	N	Valid	69	69	69	68	69
		Missing	0	0	0	1	0
	Mean		6.6812	142.4493	115.8551	70.1324	89.8406
	Median		6.0000	142.0000	115.0000	70.0000	90.0000
	Std. Deviation		2.76795	20.75809	17.93557	10.67694	13.35006
	Minimum		2.00	95.00	74.00	40.00	60.00
	Maximum		15.00	204.00	162.00	100.00	120.00
	Percentiles	25	5.0000	126.0000	105.0000	62.7500	80.0000
		50	6.0000	142.0000	115.0000	70.0000	90.0000
		75	8.5000	155.5000	125.0000	77.7500	100.0000

```
SPLIT FILE OFF.
NPAR TESTS
  /M-W= oloaika sbpmax sbpmin dbpmin dbpmax BY carTenos(0 1)
  /MISSING ANALYSIS.
```

NPar Tests

```
[DataSet1] D:\mehehu\mehehu_datat_C\tutkimus\projektit\Outi_l\Outi_K\polymorfiat.sav
```

Mann-Whitney Test

Ranks

	enos T-alleelin kantaja	N	Mean Rank	Sum of Ranks
oloaika	ei kanna T-alleelia	98	82.23	8058.50
	kantaa T-alleelia	69	86.51	5969.50
	Total	167		
sbpmax	ei kanna T-alleelia	97	79.38	7699.50
	kantaa T-alleelia	69	89.30	6161.50
	Total	166		
sbpmin	ei kanna T-alleelia	97	79.94	7754.50
	kantaa T-alleelia	69	88.50	6106.50
	Total	166		
dbpmin	ei kanna T-alleelia	97	82.25	7978.00
	kantaa T-alleelia	68	84.07	5717.00
	Total	165		
dbpmax	ei kanna T-alleelia	97	79.23	7685.50
	kantaa T-alleelia	69	89.50	6175.50
	Total	166		

Test Statistics[a]

	oloaika	sbpmax	sbpmin	dbpmin	dbpmax
Mann-Whitney U	3207.500	2946.500	3001.500	3225.000	2932.500
Wilcoxon W	8058.500	7699.500	7754.500	7978.000	7685.500
Z	-.569	-1.311	-1.131	-.242	-1.357
Asymp. Sig. (2-tailed)	.570	.190	.258	.809	.175

a. Grouping Variable: enos T-alleelin kantaja

```
****** carTestr***.
SORT CASES  BY carTestr.
SPLIT FILE LAYERED BY carTestr.


FREQUENCIES VARIABLES=oloaika sbpmax sbpmin dbpmin dbpmax
  /FORMAT=NOTABLE
  /NTILES=4
  /STATISTICS=STDDEV MINIMUM MAXIMUM MEAN MEDIAN
  /ORDER=ANALYSIS.
```

Frequencies

[DataSet1] D:\mehehu\mehehu_datat_C\tutkimus\projektit\Outi_l\Outi_K\polymorfiat.sav

Statistics

estr T-alleelin kantaja			oloaika	sbpmax	sbpmin	dbpmin	dbpmax
0	N	Valid	9	9	9	9	9
		Missing	0	0	0	0	0
	Mean		5.2222	132.8889	116.0000	71.0000	81.0000
	Median		5.0000	130.0000	115.0000	71.0000	78.0000
	Std. Deviation		1.71594	21.25702	18.03469	10.78193	11.80042
	Minimum		3.00	106.00	94.00	58.00	65.00
	Maximum		8.00	173.00	157.00	92.00	105.00
	Percentiles	25	3.5000	118.5000	103.5000	62.0000	72.5000
		50	5.0000	130.0000	115.0000	71.0000	78.0000
		75	6.5000	147.0000	119.5000	77.5000	88.0000
ei kanna T-alleelia	N	Valid	29	29	29	29	29
		Missing	0	0	0	0	0
	Mean		6.2414	143.1379	113.3103	66.7931	89.7241
	Median		7.0000	139.0000	113.0000	68.0000	88.0000
	Std. Deviation		2.06424	23.58075	20.46793	11.99815	12.85606
	Minimum		2.00	102.00	74.00	40.00	66.00
	Maximum		10.00	204.00	170.00	91.00	120.00
	Percentiles	25	4.0000	129.0000	99.5000	60.0000	83.0000
		50	7.0000	139.0000	113.0000	68.0000	88.0000
		75	7.0000	156.0000	124.5000	73.0000	100.0000
kantaa T-alleelia	N	Valid	134	133	133	132	133
		Missing	0	1	1	2	1
	Mean		6.6119	140.1955	114.4887	70.5909	87.8947
	Median		6.0000	137.0000	113.0000	70.0000	88.0000
	Std. Deviation		2.62123	19.42915	15.37738	9.31956	11.75782
	Minimum		2.00	95.00	82.00	40.00	60.00
	Maximum		15.00	210.00	162.00	100.00	113.00
	Percentiles	25	5.0000	125.5000	105.0000	64.2500	80.0000
		50	6.0000	137.0000	113.0000	70.0000	88.0000
		75	8.0000	150.0000	123.0000	78.0000	95.5000

```
SPLIT FILE OFF.
NPAR TESTS
  /M-W= oloaika sbpmax sbpmin dbpmin dbpmax BY carTestr(0 1)
  /MISSING ANALYSIS.
```

NPar Tests

[DataSet1] D:\mehehu\mehehu_datat_C\tutkimus\projektit\Outi_l\Outi_K\polymorfiat.sav

Mann-Whitney Test

Ranks

	estr T-alleelin kantaja	N	Mean Rank	Sum of Ranks
oloaika	ei kanna T-alleelia	29	79.48	2305.00
	kantaa T-alleelia	134	82.54	11061.00
	Total	163		
sbpmax	ei kanna T-alleelia	29	84.95	2463.50
	kantaa T-alleelia	133	80.75	10739.50
	Total	162		
sbpmin	ei kanna T-alleelia	29	78.55	2278.00
	kantaa T-alleelia	133	82.14	10925.00
	Total	162		
dbpmin	ei kanna T-alleelia	29	66.57	1930.50
	kantaa T-alleelia	132	84.17	11110.50
	Total	161		
dbpmax	ei kanna T-alleelia	29	87.05	2524.50
	kantaa T-alleelia	133	80.29	10678.50
	Total	162		

Test Statistics[a]

	oloaika	sbpmax	sbpmin	dbpmin	dbpmax
Mann-Whitney U	1870.000	1828.500	1843.000	1495.500	1767.500
Wilcoxon W	2305.000	10739.500	2278.000	1930.500	10678.500
Z	-.319	-.437	-.374	-1.843	-.704
Asymp. Sig. (2-tailed)	.749	.662	.709	.065	.482

a. Grouping Variable: estr T-alleelin kantaja

```
****** carCestr***.
SORT CASES  BY carCestr.
SPLIT FILE LAYERED BY carCestr.


FREQUENCIES VARIABLES=oloaika sbpmax sbpmin dbpmin dbpmax
  /FORMAT=NOTABLE
  /NTILES=4
  /STATISTICS=STDDEV MINIMUM MAXIMUM MEAN MEDIAN
  /ORDER=ANALYSIS.
```

Frequencies

```
[DataSet1] D:\mehehu\mehehu_datat_C\tutkimus\projektit\Outi_l\Outi_K\polymorfiat.sav
```

Statistics

estr C-alleelin kantaja			oloaika	sbpmax	sbpmin	dbpmin	dbpmax
.00	N	Valid	9	9	9	9	9
		Missing	0	0	0	0	0
	Mean		5.2222	132.8889	116.0000	71.0000	81.0000
	Median		5.0000	130.0000	115.0000	71.0000	78.0000
	Std. Deviation		1.71594	21.25702	18.03469	10.78193	11.80042
	Minimum		3.00	106.00	94.00	58.00	65.00
	Maximum		8.00	173.00	157.00	92.00	105.00
	Percentiles	25	3.5000	118.5000	103.5000	62.0000	72.5000
		50	5.0000	130.0000	115.0000	71.0000	78.0000
		75	6.5000	147.0000	119.5000	77.5000	88.0000
ei ole alleelin kantaja	N	Valid	52	51	51	50	51
		Missing	0	1	1	2	1
	Mean		6.5000	139.8431	113.4902	71.1800	87.3529
	Median		6.0000	136.0000	112.0000	72.0000	88.0000
	Std. Deviation		2.85945	18.86730	14.87733	8.40236	11.15854
	Minimum		2.00	107.00	86.00	54.00	67.00
	Maximum		15.00	188.00	149.00	90.00	113.00
	Percentiles	25	4.2500	125.0000	104.0000	64.2500	78.0000
		50	6.0000	136.0000	112.0000	72.0000	88.0000
		75	8.0000	148.0000	120.0000	79.0000	96.0000
alleelin kantaja	N	Valid	111	111	111	111	111
		Missing	0	0	0	0	0
	Mean		6.5676	141.1261	114.6396	69.3333	88.6216
	Median		6.0000	138.0000	113.0000	70.0000	88.0000
	Std. Deviation		2.37265	20.83446	17.01966	10.52040	12.31190
	Minimum		2.00	95.00	74.00	40.00	60.00
	Maximum		15.00	210.00	170.00	100.00	120.00
	Percentiles	25	5.0000	128.0000	104.0000	62.0000	80.0000
		50	6.0000	138.0000	113.0000	70.0000	88.0000
		75	8.0000	153.0000	124.0000	77.0000	96.0000

```
SPLIT FILE OFF.
NPAR TESTS
  /M-W= oloaika sbpmax sbpmin dbpmin dbpmax BY carCestr(0 1)
  /MISSING ANALYSIS.
```

NPar Tests

```
[DataSet1] D:\mehehu\mehehu_datat_C\tutkimus\projektit\Outi_l\Outi_K\polymorfiat.sav
```

Mann-Whitney Test

Ranks

	estr C-alleelin kantaja	N	Mean Rank	Sum of Ranks
oloaika	ei ole alleelin kantaja	52	79.78	4148.50
	alleelin kantaja	111	83.04	9217.50
	Total	163		
sbpmax	ei ole alleelin kantaja	51	80.03	4081.50
	alleelin kantaja	111	82.18	9121.50
	Total	162		
sbpmin	ei ole alleelin kantaja	51	79.94	4077.00
	alleelin kantaja	111	82.22	9126.00
	Total	162		
dbpmin	ei ole alleelin kantaja	50	87.74	4387.00
	alleelin kantaja	111	77.96	8654.00
	Total	161		
dbpmax	ei ole alleelin kantaja	51	77.93	3974.50
	alleelin kantaja	111	83.14	9228.50
	Total	162		

Test Statistics[a]

	oloaika	sbpmax	sbpmin	dbpmin	dbpmax
Mann-Whitney U	2770.500	2755.500	2751.000	2438.000	2648.500
Wilcoxon W	4148.500	4081.500	4077.000	8654.000	3974.500
Z	-.415	-.271	-.287	-1.233	-.657
Asymp. Sig. (2-tailed)	.678	.787	.774	.218	.511

a. Grouping Variable: estr C-alleelin kantaja

```
*** DIALYYSI ***.
CROSSTABS
  /TABLES=dialyysi BY enosnum inosnum estrognum carAinos carGinos carGenos carTenos carT
estr carCestr
  /FORMAT=AVALUE TABLES
  /STATISTICS=CHISQ
  /CELLS=COUNT COLUMN
  /COUNT ROUND CELL.
```

Crosstabs

```
[DataSet1] D:\mehehu\mehehu_datat_C\tutkimus\projektit\Outi_l\Outi_K\polymorfiat.sav
```

Case Processing Summary

	Cases					
	Valid		Missing		Total	
	N	Percent	N	Percent	N	Percent
dialyysi * enosnum	172	100.0%	0	0.0%	172	100.0%
dialyysi * inosnum	172	100.0%	0	0.0%	172	100.0%
dialyysi * estrogeenireseptori numeerisesti	172	100.0%	0	0.0%	172	100.0%
dialyysi * inos A-alleelin kantaja	166	96.5%	6	3.5%	172	100.0%
dialyysi * inos G-alleelin kantaja	166	96.5%	6	3.5%	172	100.0%
dialyysi * enos G-alleelin kantaja	167	97.1%	5	2.9%	172	100.0%
dialyysi * enos T-alleelin kantaja	167	97.1%	5	2.9%	172	100.0%
dialyysi * estr T-alleelin kantaja	163	94.8%	9	5.2%	172	100.0%
dialyysi * estr C-alleelin kantaja	163	94.8%	9	5.2%	172	100.0%

dialyysi * enosnum

Crosstab

			enosnum				Total
			G	Both	T	Undeterm	
dialyysi	.00	Count	94	57	9	5	165
		% within enosnum	95.9%	96.6%	90.0%	100.0%	95.9%
	1.00	Count	4	2	1	0	7
		% within enosnum	4.1%	3.4%	10.0%	0.0%	4.1%
Total		Count	98	59	10	5	172
		% within enosnum	100.0%	100.0%	100.0%	100.0%	100.0%

dialyysi * inosnum

Crosstab

			inosnum				Total
			G	Both	A	Undeterm	
dialyysi	.00	Count	102	53	4	6	165
		% within inosnum	95.3%	96.4%	100.0%	100.0%	95.9%
	1.00	Count	5	2	0	0	7
		% within inosnum	4.7%	3.6%	0.0%	0.0%	4.1%
Total		Count	107	55	4	6	172
		% within inosnum	100.0%	100.0%	100.0%	100.0%	100.0%

dialyysi * estrogeenireseptori numeerisesti

Crosstab

			estrogeenireseptori numeerisesti				Total
			T	Both	C	Undeter	
dialyysi	.00	Count	50	77	29	9	165
		% within estrogeenireseptori numeerisesti	96.2%	93.9%	100.0%	100.0%	95.9%
	1.00	Count	2	5	0	0	7
		% within estrogeenireseptori numeerisesti	3.8%	6.1%	0.0%	0.0%	4.1%
Total		Count	52	82	29	9	172
		% within estrogeenireseptori numeerisesti	100.0%	100.0%	100.0%	100.0%	100.0%

dialyysi * inos A-alleelin kantaja

Crosstab

			inos A-alleelin kantaja		Total
			ei kanna A-alleelia	kantaa A-alleelia	
dialyysi	.00	Count	102	57	159
		% within inos A-alleelin kantaja	95.3%	96.6%	95.8%
	1.00	Count	5	2	7
		% within inos A-alleelin kantaja	4.7%	3.4%	4.2%
Total		Count	107	59	166
		% within inos A-alleelin kantaja	100.0%	100.0%	100.0%

Chi-Square Tests

	Value	df	Asymp. Sig. (2-sided)	Exact Sig. (2-sided)	Exact Sig. (1-sided)
Pearson Chi-Square	.155[a]	1	.694		
Continuity Correction[b]	.000	1	1.000		
Likelihood Ratio	.160	1	.689		
Fisher's Exact Test				1.000	.519
Linear-by-Linear Association	.154	1	.695		
N of Valid Cases	166				

a. 2 cells (50.0%) have expected count less than 5. The minimum expected count is 2.49.

b. Computed only for a 2x2 table

dialyysi * inos G-alleelin kantaja

Crosstab

			inos G-alleelin kantaja		Total
			ei kanna G alleelia	kantaa G-alleelia	
dialyysi	.00	Count	4	155	159
		% within inos G-alleelin kantaja	100.0%	95.7%	95.8%
	1.00	Count	0	7	7
		% within inos G-alleelin kantaja	0.0%	4.3%	4.2%
Total		Count	4	162	166
		% within inos G-alleelin kantaja	100.0%	100.0%	100.0%

Chi-Square Tests

	Value	df	Asymp. Sig. (2-sided)	Exact Sig. (2-sided)	Exact Sig. (1-sided)
Pearson Chi-Square	.180[a]	1	.671		
Continuity Correction[b]	.000	1	1.000		
Likelihood Ratio	.349	1	.555		
Fisher's Exact Test				1.000	.840
Linear-by-Linear Association	.179	1	.672		
N of Valid Cases	166				

a. 2 cells (50.0%) have expected count less than 5. The minimum expected count is .17.

b. Computed only for a 2x2 table

dialyysi * enos G-alleelin kantaja

Crosstab

			enos G-alleelin kantaja		Total
			ei kanna G-alleelia	kantaa G-alleelia	
dialyysi	.00	Count	9	151	160
		% within enos G-alleelin kantaja	90.0%	96.2%	95.8%
	1.00	Count	1	6	7
		% within enos G-alleelin kantaja	10.0%	3.8%	4.2%
Total		Count	10	157	167
		% within enos G-alleelin kantaja	100.0%	100.0%	100.0%

Chi-Square Tests

	Value	df	Asymp. Sig. (2-sided)	Exact Sig. (2-sided)	Exact Sig. (1-sided)
Pearson Chi-Square	.894[a]	1	.345		
Continuity Correction[b]	.017	1	.895		
Likelihood Ratio	.668	1	.414		
Fisher's Exact Test				.356	.356
Linear-by-Linear Association	.888	1	.346		
N of Valid Cases	167				

a. 1 cells (25.0%) have expected count less than 5. The minimum expected count is .42.

b. Computed only for a 2x2 table

dialyysi * enos T-alleelin kantaja

Crosstab

			enos T-alleelin kantaja		
			ei kanna T-alleelia	kantaa T-alleelia	Total
dialyysi	.00	Count	94	66	160
		% within enos T-alleelin kantaja	95.9%	95.7%	95.8%
	1.00	Count	4	3	7
		% within enos T-alleelin kantaja	4.1%	4.3%	4.2%
Total		Count	98	69	167
		% within enos T-alleelin kantaja	100.0%	100.0%	100.0%

Chi-Square Tests

	Value	df	Asymp. Sig. (2-sided)	Exact Sig. (2-sided)	Exact Sig. (1-sided)
Pearson Chi-Square	.007[a]	1	.933		
Continuity Correction[b]	.000	1	1.000		
Likelihood Ratio	.007	1	.933		
Fisher's Exact Test				1.000	.612
Linear-by-Linear Association	.007	1	.933		
N of Valid Cases	167				

a. 2 cells (50.0%) have expected count less than 5. The minimum expected count is 2.89.

b. Computed only for a 2x2 table

dialyysi * estr T-alleelin kantaja

Crosstab

			estr T-alleelin kantaja		Total
			ei kanna T-alleelia	kantaa T-alleelia	
dialyysi	.00	Count	29	127	156
		% within estr T-alleelin kantaja	100.0%	94.8%	95.7%
	1.00	Count	0	7	7
		% within estr T-alleelin kantaja	0.0%	5.2%	4.3%
Total		Count	29	134	163
		% within estr T-alleelin kantaja	100.0%	100.0%	100.0%

Chi-Square Tests

	Value	df	Asymp. Sig. (2-sided)	Exact Sig. (2-sided)	Exact Sig. (1-sided)
Pearson Chi-Square	1.583[a]	1	.208		
Continuity Correction[b]	.567	1	.451		
Likelihood Ratio	2.810	1	.094		
Fisher's Exact Test				.354	.247
Linear-by-Linear Association	1.573	1	.210		
N of Valid Cases	163				

a. 1 cells (25.0%) have expected count less than 5. The minimum expected count is 1.25.

b. Computed only for a 2x2 table

dialyysi * estr C-alleelin kantaja

Crosstab

			estr C-alleelin kantaja		Total
			ei ole alleelin kantaja	alleelin kantaja	
dialyysi	.00	Count	50	106	156
		% within estr C-alleelin kantaja	96.2%	95.5%	95.7%
	1.00	Count	2	5	7
		% within estr C-alleelin kantaja	3.8%	4.5%	4.3%
Total		Count	52	111	163
		% within estr C-alleelin kantaja	100.0%	100.0%	100.0%

Chi-Square Tests

	Value	df	Asymp. Sig. (2-sided)	Exact Sig. (2-sided)	Exact Sig. (1-sided)
Pearson Chi-Square	.037[a]	1	.847		
Continuity Correction[b]	.000	1	1.000		
Likelihood Ratio	.038	1	.845		
Fisher's Exact Test				1.000	.604
Linear-by-Linear Association	.037	1	.847		
N of Valid Cases	163				

a. 2 cells (50.0%) have expected count less than 5. The minimum expected count is 2.23.

b. Computed only for a 2x2 table

```
*** DIALYYSI  puuttuvat poistettu testauksesta***.
CROSSTABS
  /TABLES=dialyysi BY enosnum inosnum estrognum
  /FORMAT=AVALUE TABLES
  /STATISTICS=CHISQ
  /CELLS=COUNT COLUMN
  /COUNT ROUND CELL.
```

Crosstabs

[DataSet1] D:\mehehu\mehehu_datat_C\tutkimus\projektit\Outi_l\Outi_K\polymorfiat.sav

Case Processing Summary

	Cases					
	Valid		Missing		Total	
	N	Percent	N	Percent	N	Percent
dialyysi * enosnum	167	97.1%	5	2.9%	172	100.0%
dialyysi * inosnum	166	96.5%	6	3.5%	172	100.0%
dialyysi * estrogeenireseptori numeerisesti	163	94.8%	9	5.2%	172	100.0%

dialyysi * enosnum

Crosstab

			enosnum			Total
			G	Both	T	
dialyysi	.00	Count	94	57	9	160
		% within enosnum	95.9%	96.6%	90.0%	95.8%
	1.00	Count	4	2	1	7
		% within enosnum	4.1%	3.4%	10.0%	4.2%
Total		Count	98	59	10	167
		% within enosnum	100.0%	100.0%	100.0%	100.0%

Chi-Square Tests

	Value	df	Asymp. Sig. (2-sided)
Pearson Chi-Square	.937[a]	2	.626
Likelihood Ratio	.717	2	.699
Linear-by-Linear Association	.190	1	.663
N of Valid Cases	167		

a. 3 cells (50.0%) have expected count less than 5. The minimum expected count is .42.

dialyysi * inosnum

Crosstab

			inosnum			Total
			G	Both	A	
dialyysi	.00	Count	102	53	4	159
		% within inosnum	95.3%	96.4%	100.0%	95.8%
	1.00	Count	5	2	0	7
		% within inosnum	4.7%	3.6%	0.0%	4.2%
Total		Count	107	55	4	166
		% within inosnum	100.0%	100.0%	100.0%	100.0%

Chi-Square Tests

	Value	df	Asymp. Sig. (2-sided)
Pearson Chi-Square	.277[a]	2	.871
Likelihood Ratio	.446	2	.800
Linear-by-Linear Association	.225	1	.635
N of Valid Cases	166		

a. 4 cells (66.7%) have expected count less than 5. The minimum expected count is .17.

dialyysi * estrogeenireseptori numeerisesti

Crosstab

			estrogeenireseptori numeerisesti			Total
			T	Both	C	
dialyysi	.00	Count	50	77	29	156
		% within estrogeenireseptori numeerisesti	96.2%	93.9%	100.0%	95.7%
	1.00	Count	2	5	0	7
		% within estrogeenireseptori numeerisesti	3.8%	6.1%	0.0%	4.3%
Total		Count	52	82	29	163
		% within estrogeenireseptori numeerisesti	100.0%	100.0%	100.0%	100.0%

Chi-Square Tests

	Value	df	Asymp. Sig. (2-sided)
Pearson Chi-Square	1.975[a]	2	.372
Likelihood Ratio	3.149	2	.207
Linear-by-Linear Association	.319	1	.572
N of Valid Cases	163		

a. 3 cells (50.0%) have expected count less than 5. The minimum expected count is 1.25.

```
*** DIALYYSI  puuttuvat poistettu testauksesta***.
CROSSTABS
  /TABLES=dialyysi BY enosnum inosnum estrognum
  /FORMAT=AVALUE TABLES
  /STATISTICS=CHISQ
  /CELLS=COUNT COLUMN
  /COUNT ROUND CELL
  /METHOD=EXACT TIMER(5).
```

Crosstabs

```
[DataSet1] D:\mehehu\mehehu_datat_C\tutkimus\projektit\Outi_l\Outi_K\polymorfiat.sav
```

Case Processing Summary

	Cases					
	Valid		Missing		Total	
	N	Percent	N	Percent	N	Percent
dialyysi * enosnum	167	97.1%	5	2.9%	172	100.0%
dialyysi * inosnum	166	96.5%	6	3.5%	172	100.0%
dialyysi * estrogeenireseptori numeerisesti	163	94.8%	9	5.2%	172	100.0%

dialyysi * enosnum

Crosstab

			enosnum			Total
			G	Both	T	
dialyysi	.00	Count	94	57	9	160
		% within enosnum	95.9%	96.6%	90.0%	95.8%
	1.00	Count	4	2	1	7
		% within enosnum	4.1%	3.4%	10.0%	4.2%
Total		Count	98	59	10	167
		% within enosnum	100.0%	100.0%	100.0%	100.0%

Chi-Square Tests

	Value	df	Asymp. Sig. (2-sided)	Exact Sig. (2-sided)	Exact Sig. (1-sided)	Point Probability
Pearson Chi-Square	.937[a]	2	.626	.631		
Likelihood Ratio	.717	2	.699	1.000		
Fisher's Exact Test	1.559			.423		
Linear-by-Linear Association	.190[b]	1	.663	.751	.431	.212
N of Valid Cases	167					

a. 3 cells (50.0%) have expected count less than 5. The minimum expected count is .42.

b. The standardized statistic is .436.

dialyysi * inosnum

Crosstab

			inosnum			Total
			G	Both	A	
dialyysi	.00	Count	102	53	4	159
		% within inosnum	95.3%	96.4%	100.0%	95.8%
	1.00	Count	5	2	0	7
		% within inosnum	4.7%	3.6%	0.0%	4.2%
Total		Count	107	55	4	166
		% within inosnum	100.0%	100.0%	100.0%	100.0%

Chi-Square Tests

	Value	df	Asymp. Sig. (2-sided)	Exact Sig. (2-sided)	Exact Sig. (1-sided)	Point Probability
Pearson Chi-Square	.277[a]	2	.871	1.000		
Likelihood Ratio	.446	2	.800	1.000		
Fisher's Exact Test	.479			1.000		
Linear-by-Linear Association	.225[b]	1	.635	.738	.479	.272
N of Valid Cases	166					

a. 4 cells (66.7%) have expected count less than 5. The minimum expected count is .17.

b. The standardized statistic is -.475.

dialyysi * estrogeenireseptori numeerisesti

Crosstab

			estrogeenireseptori numeerisesti			Total
			T	Both	C	
dialyysi	.00	Count	50	77	29	156
		% within estrogeenireseptori numeerisesti	96.2%	93.9%	100.0%	95.7%
	1.00	Count	2	5	0	7
		% within estrogeenireseptori numeerisesti	3.8%	6.1%	0.0%	4.3%
Total		Count	52	82	29	163
		% within estrogeenireseptori numeerisesti	100.0%	100.0%	100.0%	100.0%

Chi-Square Tests

	Value	df	Asymp. Sig. (2-sided)	Exact Sig. (2-sided)	Exact Sig. (1-sided)	Point Probability
Pearson Chi-Square	1.975[a]	2	.372	.402		
Likelihood Ratio	3.149	2	.207	.291		
Fisher's Exact Test	1.454			.458		
Linear-by-Linear Association	.319[b]	1	.572	.595	.392	.192
N of Valid Cases	163					

a. 3 cells (50.0%) have expected count less than 5. The minimum expected count is 1.25.

b. The standardized statistic is -.565.
