# Supplementary material for: Endothelial Nitric Oxide Synthase G894T Polymorphism Associates with Disease Severity in Puumala Hantavirus Infection
Source: PLoS One. 2015 Nov 11;10(11):e0142872. doi: 10.1371/journal.pone.0142872 (PMC4641644; doi:10.1371/journal.pone.0142872)
Supplement: S2 File — (PDF) [file pone.0142872.s002.pdf]

```
********************************************************************
* ENOS *
********************************************************************.
*** carrrier T enos   *********************.
SORT CASES  BY carTenos.
SPLIT FILE LAYERED BY carTenos.

FREQUENCIES VARIABLES=kreamax bleukmax painoero trombmin hkrmin hkrmax pil6
max crpmax
  /FORMAT=NOTABLE
  /NTILES=4
  /STATISTICS=STDDEV MINIMUM MAXIMUM MEAN MEDIAN
  /ORDER=ANALYSIS.
```

Frequencies

```
[DataSet1] G:\polymorfiat.sav
```

Statistics

enos T-alleelin kantaja			kreamax	bleukmax	painoero	trombmin
ei kanna T-alleelia	N	Valid	98	98	96	98
		Missing	0	0	2	0
	Mean		284.6735	11.0286	2.7958	70.8061
	Median		166.0000	9.5500	2.2500	65.5000
	Std. Deviation		272.19440	4.69009	2.36108	38.92331
	Minimum		51.00	4.40	.00	3.00
	Maximum		1499.00	31.20	10.40	198.00
	Percentiles	25	95.7500	7.6750	.9000	42.7500
		50	166.0000	9.5500	2.2500	65.5000
		75	381.0000	12.9250	3.8000	90.2500
kantaa T-alleelia	N	Valid	69	69	67	69
		Missing	0	0	2	0
	Mean		301.7826	11.7058	3.1149	61.8986
	Median		237.0000	10.3000	2.1000	57.0000
	Std. Deviation		263.23133	5.11595	2.97659	36.80719
	Minimum		61.00	3.90	.00	9.00
	Maximum		1285.00	26.80	12.00	238.00
	Percentiles	25	101.5000	7.7000	.8000	38.0000
		50	237.0000	10.3000	2.1000	57.0000
		75	401.5000	13.7500	4.4000	80.0000

Statistics

enos T-alleelin kantaja			hkrmin	hkrmax	pil6max	crpmax
ei kanna T-alleelia	N	Valid	98	98	62	98
		Missing	0	0	36	0
	Mean		.3564	.4379	21.0292	81.6357
	Median		.3550	.4400	14.8500	68.8500
	Std. Deviation		.04011	.05039	17.27461	51.35631
	Minimum		.25	.34	1.52	11.00
	Maximum		.46	.59	107.00	269.20
	Percentiles	25	.3300	.4000	10.5400	40.0000
		50	.3550	.4400	14.8500	68.8500
		75	.3900	.4700	26.7750	112.1250
kantaa T-alleelia	N	Valid	69	69	53	69
		Missing	0	0	16	0
	Mean		.3543	.4461	17.4808	92.4290
	Median		.3600	.4400	13.7000	84.8000
	Std. Deviation		.04268	.05849	15.46535	57.32993
	Minimum		.25	.33	1.31	16.30
	Maximum		.44	.60	96.60	266.80
	Percentiles	25	.3250	.4100	7.0900	43.1000
		50	.3600	.4400	13.7000	84.8000
		75	.3800	.4800	24.4000	125.5500

```
SPLIT FILE OFF.

NPAR TESTS
  /M-W= kreamax bleukmax painoero trombmin hkrmin hkrmax pil6max crpmax BY
carTenos(0 1)
  /MISSING ANALYSIS.
```

NPar Tests

```
[DataSet1] G:\polymorfiat.sav
```

Mann-Whitney Test

Ranks

	enos T-alleelin kantaja	N	Mean Rank	Sum of Ranks
kreamax	ei kanna T-alleelia	98	81.59	7995.50
	kantaa T-alleelia	69	87.43	6032.50
	Total	167		
bleukmax	ei kanna T-alleelia	98	81.76	8012.00
	kantaa T-alleelia	69	87.19	6016.00
	Total	167		
painoero	ei kanna T-alleelia	96	81.91	7863.50
	kantaa T-alleelia	67	82.13	5502.50
	Total	163		
trombmin	ei kanna T-alleelia	98	88.83	8705.00
	kantaa T-alleelia	69	77.14	5323.00
	Total	167		
hkrmin	ei kanna T-alleelia	98	83.91	8223.50
	kantaa T-alleelia	69	84.12	5804.50
	Total	167		
hkrmax	ei kanna T-alleelia	98	82.10	8045.50
	kantaa T-alleelia	69	86.70	5982.50
	Total	167		
pil6max	ei kanna T-alleelia	62	62.56	3878.50
	kantaa T-alleelia	53	52.67	2791.50
	Total	115		
crpmax	ei kanna T-alleelia	98	80.37	7876.50
	kantaa T-alleelia	69	89.15	6151.50
	Total	167		

Test Statistics[a]

	kreamax	bleukmax	painoero	trombmin	hkrmin	hkrmax
Mann-Whitney U	3144.500	3161.000	3207.500	2908.000	3372.500	3194.500
Wilcoxon W	7995.500	8012.000	7863.500	5323.000	8223.500	8045.500
Z	-.769	-.715	-.029	-1.537	-.028	-.607
Asymp. Sig. (2-tailed)	.442	.475	.977	.124	.978	.544

Test Statistics[a]

	pil6max	crpmax
Mann-Whitney U	1360.500	3025.500
Wilcoxon W	2791.500	7876.500
Z	-1.585	-1.155
Asymp. Sig. (2-tailed)	.113	.248

a. Grouping Variable: enos T-alleelin kantaja

```
*** carrrier G enos   *********************.
SORT CASES  BY carGenos.
SPLIT FILE LAYERED BY carGenos.

FREQUENCIES VARIABLES=kreamax bleukmax painoero trombmin hkrmin hkrmax pil6
max crpmax
  /FORMAT=NOTABLE
  /NTILES=4
  /STATISTICS=STDDEV MINIMUM MAXIMUM MEAN MEDIAN
  /ORDER=ANALYSIS.
```

Frequencies

```
[DataSet1] G:\polymorfiat.sav
```

Statistics

enos G-alleelin kantaja			kreamax	bleukmax	painoero	trombmin
ei kanna G-alleelia	N	Valid	10	10	10	10
		Missing	0	0	0	0
	Mean		478.5000	14.1800	3.8900	59.1000
	Median		325.5000	12.9000	3.5500	46.5000
	Std. Deviation		329.99369	5.85544	2.84427	46.85782
	Minimum		102.00	8.10	.00	13.00
	Maximum		1041.00	26.80	7.30	172.00
	Percentiles	25	240.0000	9.5750	1.1000	23.5000
		50	325.5000	12.9000	3.5500	46.5000
		75	816.5000	18.5000	6.8750	81.5000
kantaa G-alleelia	N	Valid	157	157	153	157
		Missing	0	0	4	0
	Mean		279.8471	11.1255	2.8641	67.6369
	Median		175.0000	9.9000	2.1000	61.0000
	Std. Deviation		260.16847	4.76078	2.61032	37.71080
	Minimum		51.00	3.90	.00	3.00
	Maximum		1499.00	31.20	12.00	238.00
	Percentiles	25	97.0000	7.6000	.9000	42.5000
		50	175.0000	9.9000	2.1000	61.0000
		75	374.5000	13.1000	3.8000	86.0000

Statistics

enos G-alleelin kantaja			hkrmin	hkrmax	pil6max	crpmax
ei kanna G-alleelia	N	Valid	10	10	9	10
		Missing	0	0	1	0
	Mean		.3420	.4840	22.6022	91.2300
	Median		.3400	.4900	13.9000	83.3500
	Std. Deviation		.05391	.06186	28.86161	70.46671
	Minimum		.25	.39	3.58	16.70
	Maximum		.42	.59	96.60	214.00
	Percentiles	25	.3050	.4275	7.5250	25.5500
		50	.3400	.4900	13.9000	83.3500
		75	.3825	.5325	24.5500	149.7750
kantaa G-alleelia	N	Valid	157	157	106	157
		Missing	0	0	51	0
	Mean		.3564	.4385	19.1214	85.7682
	Median		.3600	.4400	14.6000	75.0000
	Std. Deviation		.04019	.05236	15.19263	53.05284
	Minimum		.25	.33	1.31	11.00
	Maximum		.46	.60	107.00	269.20
	Percentiles	25	.3300	.4000	8.8600	42.4000
		50	.3600	.4400	14.6000	75.0000
		75	.3850	.4700	25.1000	118.5000

```
SPLIT FILE OFF.

NPAR TESTS
  /M-W= kreamax bleukmax painoero trombmin hkrmin hkrmax pil6max crpmax BY
carGenos(0 1)
  /MISSING ANALYSIS.
```

NPar Tests

```
[DataSet1] G:\polymorfiat.sav
```

Mann-Whitney Test

Ranks

	enos G-alleelin kantaja	N	Mean Rank	Sum of Ranks
kreamax	ei kanna G-alleelia	10	119.05	1190.50
	kantaa G-alleelia	157	81.77	12837.50
	Total	167		
bleukmax	ei kanna G-alleelia	10	113.50	1135.00
	kantaa G-alleelia	157	82.12	12893.00
	Total	167		
painoero	ei kanna G-alleelia	10	98.40	984.00
	kantaa G-alleelia	153	80.93	12382.00
	Total	163		
trombmin	ei kanna G-alleelia	10	68.10	681.00
	kantaa G-alleelia	157	85.01	13347.00
	Total	167		
hkrmin	ei kanna G-alleelia	10	69.30	693.00
	kantaa G-alleelia	157	84.94	13335.00
	Total	167		
hkrmax	ei kanna G-alleelia	10	117.20	1172.00
	kantaa G-alleelia	157	81.89	12856.00
	Total	167		
pil6max	ei kanna G-alleelia	9	53.22	479.00
	kantaa G-alleelia	106	58.41	6191.00
	Total	115		
crpmax	ei kanna G-alleelia	10	82.60	826.00
	kantaa G-alleelia	157	84.09	13202.00
	Total	167		

Test Statistics[a]

	kreamax	bleukmax	painoero	trombmin	hkrmin	hkrmax
Mann-Whitney U	434.500	490.000	601.000	626.000	638.000	453.000
Wilcoxon W	12837.500	12893.000	12382.000	681.000	693.000	12856.000
Z	-2.364	-1.990	-1.135	-1.073	-.994	-2.244
Asymp. Sig. (2-tailed)	.018	.047	.257	.283	.320	.025

Test Statistics[a]

	pil6max	crpmax
Mann-Whitney U	434.000	771.000
Wilcoxon W	479.000	826.000
Z	-.448	-.094
Asymp. Sig. (2-tailed)	.654	.925

a. Grouping Variable: enos G-alleelin kantaja

```
SPLIT FILE OFF.

*** enos  *********************.
SORT CASES  BY enosnum.
SPLIT FILE LAYERED BY enosnum.

FREQUENCIES VARIABLES=kreamax bleukmax painoero trombmin hkrmin hkrmax pil6
max crpmax
  /FORMAT=NOTABLE
  /NTILES=4
  /STATISTICS=STDDEV MINIMUM MAXIMUM MEAN MEDIAN
  /ORDER=ANALYSIS.
```

Frequencies

[DataSet1] G:\polymorfiat.sav

Statistics

enosnum			kreamax	bleukmax	painoero	trombmin
G	N	Valid	98	98	96	98
		Missing	0	0	2	0
	Mean		284.6735	11.0286	2.7958	70.8061
	Median		166.0000	9.5500	2.2500	65.5000
	Std. Deviation		272.19440	4.69009	2.36108	38.92331
	Minimum		51.00	4.40	.00	3.00
	Maximum		1499.00	31.20	10.40	198.00
	Percentiles	25	95.7500	7.6750	.9000	42.7500
		50	166.0000	9.5500	2.2500	65.5000
		75	381.0000	12.9250	3.8000	90.2500
Both	N	Valid	59	59	57	59
		Missing	0	0	2	0
	Mean		271.8305	11.2864	2.9789	62.3729
	Median		196.0000	10.2000	1.9000	57.0000
	Std. Deviation		240.92087	4.91232	3.00266	35.29965
	Minimum		61.00	3.90	.00	9.00
	Maximum		1285.00	24.00	12.00	238.00
	Percentiles	25	97.0000	7.2000	.7500	42.0000
		50	196.0000	10.2000	1.9000	57.0000
		75	369.0000	13.6000	3.8000	79.0000
T	N	Valid	10	10	10	10
		Missing	0	0	0	0
	Mean		478.5000	14.1800	3.8900	59.1000
	Median		325.5000	12.9000	3.5500	46.5000
	Std. Deviation		329.99369	5.85544	2.84427	46.85782
	Minimum		102.00	8.10	.00	13.00
	Maximum		1041.00	26.80	7.30	172.00
	Percentiles	25	240.0000	9.5750	1.1000	23.5000
		50	325.5000	12.9000	3.5500	46.5000
		75	816.5000	18.5000	6.8750	81.5000
Undeterm	N	Valid	5	5	5	5
		Missing	0	0	0	0
	Mean		163.6000	11.0800	1.7400	93.2000
	Median		143.0000	11.1000	2.0000	89.0000
	Std. Deviation		124.85311	1.80472	1.41704	21.05232
	Minimum		58.00	8.90	.40	69.00
	Maximum		368.00	13.40	3.80	127.00

Statistics

enosnum			hkrmin	hkrmax	pil6max	crpmax
G	N	Valid	98	98	62	98
		Missing	0	0	36	0
	Mean		.3564	.4379	21.0292	81.6357
	Median		.3550	.4400	14.8500	68.8500
	Std. Deviation		.04011	.05039	17.27461	51.35631
	Minimum		.25	.34	1.52	11.00
	Maximum		.46	.59	107.00	269.20
	Percentiles	25	.3300	.4000	10.5400	40.0000
		50	.3550	.4400	14.8500	68.8500
		75	.3900	.4700	26.7750	112.1250
Both	N	Valid	59	59	44	59
		Missing	0	0	15	0
	Mean		.3564	.4397	16.4332	92.6322
	Median		.3600	.4300	12.3700	85.9000
	Std. Deviation		.04067	.05592	11.29820	55.52107
	Minimum		.25	.33	1.31	16.30
	Maximum		.44	.60	54.74	266.80
	Percentiles	25	.3300	.4000	7.0050	45.0000
		50	.3600	.4300	12.3700	85.9000
		75	.3800	.4700	24.6500	124.1000
T	N	Valid	10	10	9	10
		Missing	0	0	1	0
	Mean		.3420	.4840	22.6022	91.2300
	Median		.3400	.4900	13.9000	83.3500
	Std. Deviation		.05391	.06186	28.86161	70.46671
	Minimum		.25	.39	3.58	16.70
	Maximum		.42	.59	96.60	214.00
	Percentiles	25	.3050	.4275	7.5250	25.5500
		50	.3400	.4900	13.9000	83.3500
		75	.3825	.5325	24.5500	149.7750
Undeterm	N	Valid	5	5	3	5
		Missing	0	0	2	0
	Mean		.3800	.4300	15.9167	63.6400
	Median		.3800	.4200	12.5000	75.0000
	Std. Deviation		.02000	.04062	6.10936	37.09809
	Minimum		.36	.39	12.28	22.00
	Maximum		.40	.49	22.97	102.60

Statistics

enosnum			kreamax	bleukmax	painoero	trombmin
Undeterm	Percentiles	25	64.0000	9.3500	.4000	78.5000
		50	143.0000	11.1000	2.0000	89.0000
		75	273.5000	12.8000	2.9500	110.0000

Statistics

enosnum			hkrmin	hkrmax	pil6max	crpmax
Undeterm	Percentiles	25	.3600	.3950	12.2800	24.5000
		50	.3800	.4200	12.5000	75.0000
		75	.4000	.4700	.	97.1000

```
SPLIT FILE OFF.
NPAR TESTS
  /K-W=kreamax bleukmax painoero trombmin hkrmin hkrmax pil6max crpmax BY e
nosnum(1 3)
  /MISSING ANALYSIS.
```

NPar Tests

```
[DataSet1] G:\polymorfiat.sav
```

Kruskal-Wallis Test

Ranks

	enosnum	N	Mean Rank
kreamax	G	98	81.59
	Both	59	82.07
	T	10	119.05
	Total	167	
bleukmax	G	98	81.76
	Both	59	82.73
	T	10	113.50
	Total	167	
painoero	G	96	81.91
	Both	57	79.27
	T	10	98.40
	Total	163	
trombmin	G	98	88.83
	Both	59	78.68
	T	10	68.10
	Total	167	
hkrmin	G	98	83.91
	Both	59	86.64
	T	10	69.30
	Total	167	
hkrmax	G	98	82.10
	Both	59	81.53
	T	10	117.20
	Total	167	
pil6max	G	62	62.56
	Both	44	52.56
	T	9	53.22
	Total	115	
crpmax	G	98	80.37
	Both	59	90.26
	T	10	82.60
	Total	167	

Test Statistics[a,b]

	kreamax	bleukmax	painoero	trombmin	hkrmin	hkrmax
Chi-Square	5.593	3.975	1.399	2.773	1.106	5.039
df	2	2	2	2	2	2
Asymp. Sig.	.061	.137	.497	.250	.575	.081

Test Statistics[a,b]

	pil6max	crpmax
Chi-Square	2.515	1.550
df	2	2
Asymp. Sig.	.284	.461

a. Kruskal Wallis Test
b. Grouping Variable: enosnum
