# Supplementary material for: Endothelial Nitric Oxide Synthase G894T Polymorphism Associates with Disease Severity in Puumala Hantavirus Infection
Source: PLoS One. 2015 Nov 11;10(11):e0142872. doi: 10.1371/journal.pone.0142872 (PMC4641644; doi:10.1371/journal.pone.0142872)
Supplement: S3 File — (PDF) [file pone.0142872.s003.pdf]

```
GET
  FILE='G:\polymorfiat.sav'.
DATASET NAME DataSet1 WINDOW=FRONT.
FREQUENCIES VARIABLES=carAinos carGinos inosnum carGenos carTenos enosnum
  /ORDER=ANALYSIS.
```

Frequencies

[DataSet1] G:\polymorfiat.sav

Statistics

		inos A-alleelin kantaja	inos G-alleelin kantaja	inosnum	enos G-alleelin kantaja
N	Valid	166	166	172	167
	Missing	6	6	0	5

Statistics

		enos T-alleelin kantaja	enosnum
N	Valid	167	172
	Missing	5	0

Frequency Table

inos A-alleelin kantaja

		Frequency	Percent	Valid Percent	Cumulative Percent
Valid	ei kanna A-alleelia	107	62.2	64.5	64.5
	kantaa A-alleelia	59	34.3	35.5	100.0
	Total	166	96.5	100.0	
Missing	System	6	3.5		
Total		172	100.0		

inos G-alleelin kantaja

		Frequency	Percent	Valid Percent	Cumulative Percent
Valid	ei kanna G alleelia	4	2.3	2.4	2.4
	kantaa G-alleelia	162	94.2	97.6	100.0
	Total	166	96.5	100.0	
Missing	System	6	3.5		
Total		172	100.0		

inosnum

		Frequency	Percent	Valid Percent	Cumulative Percent
Valid	G	107	62.2	62.2	62.2
	Both	55	32.0	32.0	94.2
	A	4	2.3	2.3	96.5
	Undeterm	6	3.5	3.5	100.0
	Total	172	100.0	100.0	

enos G-alleelin kantaja

		Frequency	Percent	Valid Percent	Cumulative Percent
Valid	ei kanna G-alleelia	10	5.8	6.0	6.0
	kantaa G-alleelia	157	91.3	94.0	100.0
	Total	167	97.1	100.0	
Missing	System	5	2.9		
Total		172	100.0		

enos T-alleelin kantaja

		Frequency	Percent	Valid Percent	Cumulative Percent
Valid	ei kanna T-alleelia	98	57.0	58.7	58.7
	kantaa T-alleelia	69	40.1	41.3	100.0
	Total	167	97.1	100.0	
Missing	System	5	2.9		
Total		172	100.0		

enosnum

		Frequency	Percent	Valid Percent	Cumulative Percent
Valid	G	98	57.0	57.0	57.0
	Both	59	34.3	34.3	91.3
	T	10	5.8	5.8	97.1
	Undeterm	5	2.9	2.9	100.0
	Total	172	100.0	100.0	

```
FREQUENCIES VARIABLES=carTestr carCestr estrognum
  /ORDER=ANALYSIS.
```

Frequencies

```
[DataSet1] G:\polymorfiat.sav
```

Statistics

		estr T-alleelin kantaja	estr C-alleelin kantaja	estrogeenireseptori numeerisesti
N	Valid	163	163	172
	Missing	9	9	0

Frequency Table

estr T-alleelin kantaja

		Frequency	Percent	Valid Percent	Cumulative Percent
Valid	ei kanna T-alleelia	29	16.9	17.8	17.8
	kantaa T-alleelia	134	77.9	82.2	100.0
	Total	163	94.8	100.0	
Missing	System	9	5.2		
Total		172	100.0		

estr C-alleelin kantaja

		Frequency	Percent	Valid Percent	Cumulative Percent
Valid	ei ole alleelin kantaja	52	30.2	31.9	31.9
	alleelin kantaja	111	64.5	68.1	100.0
	Total	163	94.8	100.0	
Missing	System	9	5.2		
Total		172	100.0		

estrogeenireseptori numeerisesti

		Frequency	Percent	Valid Percent	Cumulative Percent
Valid	T	52	30.2	30.2	30.2
	Both	82	47.7	47.7	77.9
	C	29	16.9	16.9	94.8
	Undeter	9	5.2	5.2	100.0
	Total	172	100.0	100.0	

```
*** carrrier A inos   *********************.
SORT CASES  BY carAinos.
SPLIT FILE LAYERED BY carAinos.

FREQUENCIES VARIABLES=kreamax bleukmax painoero trombmin hkrmin hkrmax pil6
max crpmax
  /FORMAT=NOTABLE
  /NTILES=4
  /STATISTICS=STDDEV MINIMUM MAXIMUM MEAN MEDIAN
  /ORDER=ANALYSIS.
```

Frequencies

```
[DataSet1] G:\polymorfiat.sav
```

Statistics

inos A-alleelin kantaja			kreamax	bleukmax	painoero	trombmin
ei kanna A-alleelia	N	Valid	107	107	104	107
		Missing	0	0	3	0
	Mean		306.9626	11.4327	3.0096	70.1495
	Median		213.0000	10.3000	2.1500	65.0000
	Std. Deviation		273.17369	4.71829	2.60617	41.48398
	Minimum		51.00	3.90	.00	9.00
	Maximum		1285.00	26.80	12.00	238.00
	Percentiles	25	103.0000	8.1000	.9000	41.0000
		50	213.0000	10.3000	2.1500	65.0000
		75	445.0000	13.5000	4.0750	90.0000
kantaa A-alleelia	N	Valid	59	59	58	59
		Missing	0	0	1	0
	Mean		261.8136	10.8814	2.7914	64.5254
	Median		163.0000	8.9000	2.1000	57.0000
	Std. Deviation		260.90000	5.05796	2.69589	31.95274
	Minimum		52.00	5.10	.00	3.00
	Maximum		1499.00	31.20	10.40	159.00
	Percentiles	25	85.0000	7.5000	.6750	43.0000
		50	163.0000	8.9000	2.1000	57.0000
		75	359.0000	12.6000	3.5500	81.0000

Statistics

inos A-alleelin kantaja			hkrmin	hkrmax	pil6max	crpmax
ei kanna A-alleelia	N	Valid	107	107	76	107
		Missing	0	0	31	0
	Mean		.3587	.4459	19.1736	84.2551
	Median		.3600	.4400	13.5000	72.7000
	Std. Deviation		.04028	.05625	18.42707	51.68977
	Minimum		.25	.33	1.31	11.00
	Maximum		.46	.60	107.00	239.80
	Percentiles	25	.3300	.4000	8.2450	42.7000
		50	.3600	.4400	13.5000	72.7000
		75	.3900	.4800	23.4400	118.0000
kantaa A-alleelia	N	Valid	59	59	37	59
		Missing	0	0	22	0
	Mean		.3515	.4322	20.1543	90.0237
	Median		.3500	.4300	17.2000	79.0000
	Std. Deviation		.04338	.04724	12.26078	57.93077
	Minimum		.25	.34	4.39	15.90
	Maximum		.44	.59	44.81	269.20
	Percentiles	25	.3200	.4000	8.8400	35.7000
		50	.3500	.4300	17.2000	79.0000
		75	.3900	.4600	27.4800	120.0000

```
SPLIT FILE OFF.

NPAR TESTS
  /M-W= kreamax bleukmax painoero trombmin hkrmin hkrmax pil6max crpmax BY
carAinos(0 1)
  /MISSING ANALYSIS.
```

NPar Tests

```
[DataSet1] G:\polymorfiat.sav
```

Mann-Whitney Test

Ranks

	inos A-alleelin kantaja	N	Mean Rank	Sum of Ranks
kreamax	ei kanna A-alleelia	107	88.07	9423.00
	kantaa A-alleelia	59	75.22	4438.00
	Total	166		
bleukmax	ei kanna A-alleelia	107	87.07	9316.50
	kantaa A-alleelia	59	77.03	4544.50
	Total	166		
painoero	ei kanna A-alleelia	104	83.81	8716.50
	kantaa A-alleelia	58	77.35	4486.50
	Total	162		
trombmin	ei kanna A-alleelia	107	85.16	9112.00
	kantaa A-alleelia	59	80.49	4749.00
	Total	166		
hkrmin	ei kanna A-alleelia	107	85.56	9155.00
	kantaa A-alleelia	59	79.76	4706.00
	Total	166		
hkrmax	ei kanna A-alleelia	107	87.83	9398.00
	kantaa A-alleelia	59	75.64	4463.00
	Total	166		
pil6max	ei kanna A-alleelia	76	54.41	4135.50
	kantaa A-alleelia	37	62.31	2305.50
	Total	113		
crpmax	ei kanna A-alleelia	107	82.29	8805.00
	kantaa A-alleelia	59	85.69	5056.00
	Total	166		

Test Statistics[a]

	kreamax	bleukmax	painoero	trombmin	hkrmin	hkrmax
Mann-Whitney U	2668.000	2774.500	2775.500	2979.000	2936.000	2693.000
Wilcoxon W	4438.000	4544.500	4486.500	4749.000	4706.000	4463.000
Z	-1.648	-1.289	-.841	-.599	-.746	-1.567
Asymp. Sig. (2-tailed)	.099	.197	.401	.549	.456	.117

Test Statistics[a]

	pil6max	crpmax
Mann-Whitney U	1209.500	3027.000
Wilcoxon W	4135.500	8805.000
Z	-1.202	-.437
Asymp. Sig. (2-tailed)	.229	.662

a. Grouping Variable: inos A-alleelin kantaja

```
*** carrrier G inos   *********************.
SORT CASES  BY carGinos.
SPLIT FILE LAYERED BY carGinos.

FREQUENCIES VARIABLES=kreamax bleukmax painoero trombmin hkrmin hkrmax pil6
max crpmax
  /FORMAT=NOTABLE
  /NTILES=4
  /STATISTICS=STDDEV MINIMUM MAXIMUM MEAN MEDIAN
  /ORDER=ANALYSIS.
```

Frequencies

```
[DataSet1] G:\polymorfiat.sav
```

Statistics

inos G-alleelin kantaja			kreamax	bleukmax	painoero	trombmin
ei kanna G alleelia	N	Valid	4	4	4	4
		Missing	0	0	0	0
	Mean		153.7500	9.8500	1.8250	44.7500
	Median		83.0000	8.4000	1.8000	52.0000
	Std. Deviation		153.78855	5.36252	1.51959	32.50000
	Minimum		65.00	5.10	.00	3.00
	Maximum		384.00	17.50	3.70	72.00
	Percentiles	25	68.2500	5.7500	.4000	11.0000
		50	83.0000	8.4000	1.8000	52.0000
		75	310.0000	15.4000	3.2750	71.2500
kantaa G-alleelia	N	Valid	162	162	158	162
		Missing	0	0	4	0
	Mean		294.3025	11.2710	2.9595	68.7284
	Median		193.5000	10.0000	2.2000	62.0000
	Std. Deviation		270.58224	4.83325	2.65138	38.40161
	Minimum		51.00	3.90	.00	9.00
	Maximum		1499.00	31.20	12.00	238.00
	Percentiles	25	98.7500	7.6750	.9000	42.0000
		50	193.5000	10.0000	2.2000	62.0000
		75	381.5000	13.1000	3.9250	88.0000

Statistics

inos G-alleelin kantaja			hkrmin	hkrmax	pil6max	crpmax
ei kanna G alleelia	N	Valid	4	4	2	4
		Missing	0	0	2	0
	Mean		.3850	.4600	11.4150	42.9750
	Median		.3900	.4500	11.4150	43.5000
	Std. Deviation		.02646	.05033	4.27800	24.07341
	Minimum		.35	.41	8.39	15.90
	Maximum		.41	.53	14.44	69.00
	Percentiles	25	.3575	.4200	8.3900	19.6000
		50	.3900	.4500	11.4150	43.5000
		75	.4075	.5100	.	65.8250
kantaa G-alleelia	N	Valid	162	162	111	162
		Missing	0	0	51	0
	Mean		.3554	.4406	19.6403	87.3753
	Median		.3600	.4400	14.6000	78.9500
	Std. Deviation		.04153	.05362	16.71996	54.01242
	Minimum		.25	.33	1.31	11.00
	Maximum		.46	.60	107.00	269.20
	Percentiles	25	.3300	.4000	8.7700	42.5500
		50	.3600	.4400	14.6000	78.9500
		75	.3900	.4700	25.4000	120.0000

```
SPLIT FILE OFF.

NPAR TESTS
  /M-W= kreamax bleukmax painoero trombmin hkrmin hkrmax pil6max crpmax BY
carGinos(0 1)
  /MISSING ANALYSIS.
```

NPar Tests

```
[DataSet1] G:\polymorfiat.sav
```

Mann-Whitney Test

Ranks

	inos G-alleelin kantaja	N	Mean Rank	Sum of Ranks
kreamax	ei kanna G alleelia	4	44.25	177.00
	kantaa G-alleelia	162	84.47	13684.00
	Total	166		
bleukmax	ei kanna G alleelia	4	65.63	262.50
	kantaa G-alleelia	162	83.94	13598.50
	Total	166		
painoero	ei kanna G alleelia	4	63.88	255.50
	kantaa G-alleelia	158	81.95	12947.50
	Total	162		
trombmin	ei kanna G alleelia	4	57.25	229.00
	kantaa G-alleelia	162	84.15	13632.00
	Total	166		
hkrmin	ei kanna G alleelia	4	121.00	484.00
	kantaa G-alleelia	162	82.57	13377.00
	Total	166		
hkrmax	ei kanna G alleelia	4	101.63	406.50
	kantaa G-alleelia	162	83.05	13454.50
	Total	166		
pil6max	ei kanna G alleelia	2	42.00	84.00
	kantaa G-alleelia	111	57.27	6357.00
	Total	113		
crpmax	ei kanna G alleelia	4	39.25	157.00
	kantaa G-alleelia	162	84.59	13704.00
	Total	166		

Test Statistics[b]

	kreamax	bleukmax	painoero	trombmin	hkrmin	hkrmax
Mann-Whitney U	167.000	252.500	245.500	219.000	174.000	251.500
Wilcoxon W	177.000	262.500	255.500	229.000	13377.000	13454.500
Z	-1.653	-.753	-.761	-1.106	-1.584	-.765
Asymp. Sig. (2-tailed)	.098	.451	.447	.269	.113	.444
Exact Sig. [2*(1-tailed Sig.)]						

Test Statistics[b]

	pil6max	crpmax
Mann-Whitney U	81.000	147.000
Wilcoxon W	84.000	157.000
Z	-.653	-1.864
Asymp. Sig. (2-tailed)	.514	.062
Exact Sig. [2*(1-tailed Sig.)]	.544[a]	

a. Not corrected for ties.
b. Grouping Variable: inos G-alleelin kantaja

```
*** inos  ********************.
SORT CASES  BY inosnum.
SPLIT FILE LAYERED BY inosnum.

FREQUENCIES VARIABLES=kreamax bleukmax painoero trombmin hkrmin hkrmax pil6
max crpmax
  /FORMAT=NOTABLE
  /NTILES=4
  /STATISTICS=STDDEV MINIMUM MAXIMUM MEAN MEDIAN
  /ORDER=ANALYSIS.
```

Frequencies

```
[DataSet1] G:\polymorfiat.sav
```

Statistics

inosnum			kreamax	bleukmax	painoero	trombmin
G	N	Valid	107	107	104	107
		Missing	0	0	3	0
	Mean		306.9626	11.4327	3.0096	70.1495
	Median		213.0000	10.3000	2.1500	65.0000
	Std. Deviation		273.17369	4.71829	2.60617	41.48398
	Minimum		51.00	3.90	.00	9.00
	Maximum		1285.00	26.80	12.00	238.00
	Percentiles	25	103.0000	8.1000	.9000	41.0000
		50	213.0000	10.3000	2.1500	65.0000
		75	445.0000	13.5000	4.0750	90.0000
Both	N	Valid	55	55	54	55
		Missing	0	0	1	0
	Mean		269.6727	10.9564	2.8630	65.9636
	Median		166.0000	8.9000	2.2000	57.0000
	Std. Deviation		266.21246	5.07896	2.75861	31.73091
	Minimum		52.00	5.60	.00	17.00
	Maximum		1499.00	31.20	10.40	159.00
	Percentiles	25	88.0000	7.5000	.6750	45.0000
		50	166.0000	8.9000	2.2000	57.0000
		75	359.0000	12.6000	3.5750	83.0000
A	N	Valid	4	4	4	4
		Missing	0	0	0	0
	Mean		153.7500	9.8500	1.8250	44.7500
	Median		83.0000	8.4000	1.8000	52.0000
	Std. Deviation		153.78855	5.36252	1.51959	32.50000
	Minimum		65.00	5.10	.00	3.00
	Maximum		384.00	17.50	3.70	72.00
	Percentiles	25	68.2500	5.7500	.4000	11.0000
		50	83.0000	8.4000	1.8000	52.0000
		75	310.0000	15.4000	3.2750	71.2500
Undeterm	N	Valid	6	6	6	6
		Missing	0	0	0	0
	Mean		207.8333	13.1000	1.8167	60.5000
	Median		201.5000	13.1500	1.7000	58.5000
	Std. Deviation		123.92484	3.74273	1.44418	28.95341
	Minimum		70.00	8.20	.20	15.00
	Maximum		401.00	18.20	3.70	93.00

Statistics

inosnum			hkrmin	hkrmax	pil6max	crpmax
G	N	Valid	107	107	76	107
		Missing	0	0	31	0
	Mean		.3587	.4459	19.1736	84.2551
	Median		.3600	.4400	13.5000	72.7000
	Std. Deviation		.04028	.05625	18.42707	51.68977
	Minimum		.25	.33	1.31	11.00
	Maximum		.46	.60	107.00	239.80
	Percentiles	25	.3300	.4000	8.2450	42.7000
		50	.3600	.4400	13.5000	72.7000
		75	.3900	.4800	23.4400	118.0000
Both	N	Valid	55	55	35	55
		Missing	0	0	20	0
	Mean		.3491	.4302	20.6537	93.4455
	Median		.3500	.4300	21.6000	85.9000
	Std. Deviation		.04352	.04684	12.40491	58.27928
	Minimum		.25	.34	4.39	16.30
	Maximum		.44	.59	44.81	269.20
	Percentiles	25	.3200	.3900	8.9100	41.4000
		50	.3500	.4300	21.6000	85.9000
		75	.3900	.4600	27.5600	127.0000
A	N	Valid	4	4	2	4
		Missing	0	0	2	0
	Mean		.3850	.4600	11.4150	42.9750
	Median		.3900	.4500	11.4150	43.5000
	Std. Deviation		.02646	.05033	4.27800	24.07341
	Minimum		.35	.41	8.39	15.90
	Maximum		.41	.53	14.44	69.00
	Percentiles	25	.3575	.4200	8.3900	19.6000
		50	.3900	.4500	11.4150	43.5000
		75	.4075	.5100	.	65.8250
Undeterm	N	Valid	6	6	5	6
		Missing	0	0	1	0
	Mean		.3600	.4383	15.0280	61.5667
	Median		.3600	.4500	14.2000	51.5000
	Std. Deviation		.01673	.05811	5.29451	43.20063
	Minimum		.33	.35	8.89	20.00
	Maximum		.38	.50	22.97	118.80

Statistics

inosnum			kreamax	bleukmax	painoero	trombmin
Undeterm	Percentiles	25	97.7500	9.4000	.3500	40.5000
		50	201.5000	13.1500	1.7000	58.5000
		75	299.7500	16.6250	3.3250	90.0000

Statistics

inosnum			hkrmin	hkrmax	pil6max	crpmax
Undeterm	Percentiles	25	.3525	.3800	10.5850	23.7500
		50	.3600	.4500	14.2000	51.5000
		75	.3725	.4925	19.8850	106.6500

```
NPAR TESTS
  /K-W=kreamax bleukmax painoero trombmin hkrmin hkrmax pil6max crpmax BY i
nosnum(1 3)
  /MISSING ANALYSIS.
```

NPar Tests

```
[DataSet1] G:\polymorfiat.sav
```

Kruskal-Wallis Test

Ranks

	inosnum	N	Mean Rank
kreamax	G	107	88.07
	Both	55	77.47
	A	4	44.25
	Total	166	
bleukmax	G	107	87.07
	Both	55	77.85
	A	4	65.63
	Total	166	
painoero	G	104	83.81
	Both	54	78.35
	A	4	63.88
	Total	162	
trombmin	G	107	85.16
	Both	55	82.18
	A	4	57.25
	Total	166	
hkrmin	G	107	85.56
	Both	55	76.76
	A	4	121.00
	Total	166	
hkrmax	G	107	87.83
	Both	55	73.75
	A	4	101.63
	Total	166	
pil6max	G	76	54.41
	Both	35	63.47
	A	2	42.00
	Total	113	
crpmax	G	107	82.29
	Both	55	89.07
	A	4	39.25
	Total	166	

Test Statistics[a,b]

	kreamax	bleukmax	painoero	trombmin	hkrmin	hkrmax
Chi-Square	4.498	1.903	1.061	1.362	3.733	3.713
df	2	2	2	2	2	2
Asymp. Sig.	.106	.386	.588	.506	.155	.156

Test Statistics[a,b]

	pil6max	crpmax
Chi-Square	2.258	4.198
df	2	2
Asymp. Sig.	.323	.123

a. Kruskal Wallis Test
b. Grouping Variable: inosnum
